# A simulation model to investigate interactions between first season grazing calves and *Ostertagia ostertagi*

**DOI:** 10.1016/j.vetpar.2016.05.001

**Published:** 2016-08-15

**Authors:** Zoe Berk, Stephen C. Bishop, Andrew B. Forbes, Ilias Kyriazakis

**Affiliations:** a School of Agriculture Food and Rural Development, Newcastle University, Newcastle upon Tyne, NE1 7RU, UK; b The Roslin Institute and Royal (Dick) School of Veterinary Studies, University of Edinburgh, Midlothian, EH25 9RG, Scotland, UK; c Scottish Centre for Production Animal Health and Food Safety, School of Veterinary Medicine, University of Glasgow, G61 1QH, Scotland, UK

**Keywords:** Calves, Gastrointestinal parasites, Immunity, Modelling, *Ostertagia ostertagi*, Parasite-induced anorexia

## Abstract

A dynamic, deterministic model was developed to investigate the consequences of parasitism with *Ostertagia ostertagi*, the most prevalent and economically important gastrointestinal parasite of cattle in temperate regions. Interactions between host and parasite were considered to predict the level of parasitism and performance of an infected calf. Key model inputs included calf intrinsic growth rate, feed quality and mode and level of infection. The effects of these varied inputs were simulated on a daily basis for key parasitological (worm burden, total egg output and faecal egg count) and performance outputs (feed intake and bodyweight) over a 6 month grazing period. Data from published literature were used to parameterise the model and its sensitivity was tested for uncertain parameters by a Latin hypercube sensitivity design. For the latter each parameter tested was subject to a 20% coefficient of variation. The model parasitological outputs were most sensitive to the immune rate parameters that affected overall worm burdens. The model predicted the expected larger worm burdens along with disproportionately greater body weight losses with increasing daily infection levels. The model was validated against published literature using graphical and statistical comparisons. Its predictions were quantitatively consistent with the parasitological outputs of published experiments in which calves were subjected to different infection levels. The consequences of model weaknesses are discussed and point towards model improvements. Future work should focus on developing a stochastic model to account for calf variation in performance and immune response; this will ultimately be used to test the effectiveness of different parasite control strategies in naturally infected calf populations.

## Highlights

• A deterministic model to address calf*—O. ostertagi* interactions was developed.
• The model predicts performance and FEC for different infection intensities.
• It performs well when validated against published data.
• It does not account for calf genotypic variation.
• A future aim is to develop a stochastic model to account for between host variation.

## 1 Introduction

There are increased concerns about prospects for sustainable control of gastrointestinal parasites in grazing ruminants. These stem from a variety of risks, including the loss of infection resistance as hosts are selected for production intensity (Mackinnon et al., 1991), climate change effects on parasite dynamics (Skuce et al., 2013), and the increased incidence of parasite resistance to anthelmintics (Rose et al., 2015). Although the latter has been more commonly identified for small ruminants, there is increasing evidence that it is also happening for cattle (Edmonds et al., 2010, O’shaughnessy et al., 2014). Amongst others, Sutherland and Leathwick (2011) have reported parasite resistance to the three broad-spectrum anthelmintic classes (benzimidazoles, levamisole and macrocyclic lactones) used on cattle.

For this reason there is a need to develop strategies that would enable sustainable control of gastrointestinal parasites and maintain the effectiveness of chemoprophylaxis (Charlier et al., 2014). Several strategies that may achieve this have been proposed, including targeted selective treatment (TST), breeding cattle resistant to parasites and grazing management. Testing for the effectiveness and interactions of such strategies is very difficult both experimentally and in practice. This is due to cost and difficulties in making fair comparisons, in the absence of confounding variables; for example although traits have been independently evaluated for TST in cattle, a direct comparison with other applied control strategies has not yet been conducted (Höglund et al., 2009, Höglund et al., 2013).

Recently, simulation models have been used to make such direct comparisons for control strategies on parasitised sheep (Laurenson et al., 2012a, Laurenson et al., 2013a, Laurenson et al., 2013b). Investigating the consequences of such strategies in silico for cattle may be one cost effective and time efficient way of overcoming the above limitations. Currently there are only two simulation models which investigate host-parasite interactions for cattle (Smith et al., 1987, Ward, 2006a). Both models have their limitations; for example, the former model cannot make predictions about the consequences of parasitism on performance, whereas the latter uses bodyweight as the only descriptor of the animal. The objective of this paper was to develop a novel simulation model to account for the interactions between *Ostertagia ostertagi*, the most prevalent parasite of cattle worldwide, particularly in temperate regions (Tisdell et al., 1999), and immunologically naïve calves, which are most at risk from parasitism. Emphasis in model development was given to accounting for within host parasite dynamics and their effects on host performance. The model was developed with the view of introducing between-animal variation in later steps.

## 2 Materials and methods

### 2.1 Model development

The model stems from the approach of Laurenson et al. (2011) to simulate the effects of *Teladorsagia circumcincta* challenge on growing lambs. The developed model is deterministic and dynamic, as it predicts the responses of a single calf to infection over time.

#### 2.1.1 Parasite-free animal

##### 2.1.1.1 Basic intrinsic growth model

The calf considered was a weaned, castrated male (steer) Limousin X Holstein Friesian born in autumn; this common cross currently represents the majority of beef cattle in the UK (Todd et al., 2011). Autumn born calves are capable of utilising grass in spring and hence are turned out at approximately 6 months of age and left at pasture until late autumn (Phillips, 2010).

The empty body mass composition of a calf comprises of its components protein, lipid, ash, water and a negligible amount of carbohydrates; each of these have an expected growth rate (Supplementary Data S1) defined by animal genotype (Emmans and Kyriazakis, 2001). According to Wellock et al. (2004) intrinsic growth of mammals can be modelled using a sigmoidal growth function, where the calves grow at a rate relative to their current and mature mass. Thus in order to predict intrinsic, henceforth called ‘desired’, growth, only three parameters were required: the current body mass of the animal, its growth rate parameter *B* (day^−1^) and its mature body mass (Emmans and Kyriazakis, 1997). It was further assumed that the animal has an intrinsic body fatness, which was defined by the lipid to protein ratio at maturity (Emmans, 1997). The mature empty body mass (*EBW_M_*) was estimated at 680 kg and the *B* rate parameter as 0.0071 day^−1^ for steers from the data of English Beef and Lamb Executive (EBLEX) Better Returns Programme (2005) (Supplementary Data S1). The total bodyweight (*BW*) of the calf at any given time point was the sum of the empty body weight and the gutfill (*GF*) of the calf.

##### 2.1.1.2 Resource requirements and feed intake

As with previous models (Vagenas et al., 2007a Laurenson et al., 2011) only protein and energy requirements were considered, as all other nutrient requirements were assumed to be fulfilled by the feed and were not limiting to the calf (Wellock et al., 2004). It is generally accepted that healthy ruminants allocate feed resources to three functions: maintenance, growth and reproduction (Coop and Kyriazakis, 1999). Equations for the protein and energy requirements for the processes of maintenance and growth are given in Supplementary Data S1.

It was assumed that the calf attempts to eat to fulfil its requirements for the first limiting feed resource (Emmans and Kyriazakis, 2001). As feed quality declines, feed intake initially increases, to a maximum defined by gut capacity (Kyriazakis and Emmans, 1995). Hence feed bulk is the only constraint that may prevent a healthy calf from satisfying its requirement. Equations to describe the feed intake needed to fulfil protein and energy requirements are given in Supplementary Data S1. In order to reflect the day to day variation in calf feed intake, a random effect caused by environmental influences was assumed (Doeschl-Wilson et al., 2008).

##### 2.1.1.3 Allocation of constrained resources

There are numerous circumstances under which intake of resources may be insufficient to meet the needs of all primary functions (requirements). When this happens, the animal has the problem of how to allocate its limiting feed resources (Coop and Kyriazakis, 1999). Here, it was assumed that the requirements for maintenance were met first, and any excess was allocated to growth. The efficiency of protein deposition and lipid deposition were considered to be 0.50 and 0.59, respectively (AFRC, 1993). If there are insufficient resources to fulfil maintenance requirements then the host will undergo catabolism of protein and lipid body reserves and ensure calf survival in the short-run. If either of these deficiencies is maintained over a significant time period the calf will continue to catabolise stores until death occurs.

#### 2.1.2 Parasitised calf

**Fig. 1:**
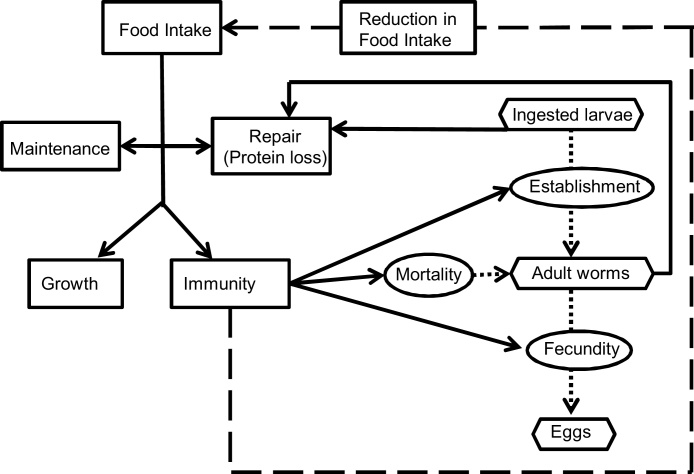
A schematic description of the parasite-host interactions. The rectangular boxes and solid lines indicate the flow of ingested feed resources; the oval boxes indicate the host-parasite interactions and the hexagonal boxes represent the key measurabe stages of the parasite life-cycle. Host immune response is assumed to lead to parasite-induced anorexia (broken line).

The model describes the host-parasite interactions presented in Fig. 1. The process starts with the ingestion of larvae, a proportion of which will establish in the gastrointestinal tract and develop into adult worms resulting in a cost to the host in terms of protein loss (Fox, 1993). Of these adult worms a proportion will die on each given day and any surviving adult female will produce eggs. These three processes are affected by the host through its immune responses.

##### 2.1.2.1 Immune response

Calves were assumed to have had no prior parasitic exposure at turnout to pasture. Although the immune response to *O. ostertagi* is currently not well understood (Li et al., 2010), worm burden has been found to show significant negative correlation to level of parasitic exposure over time (Vercruysse and Claerebout, 1997). Immune development following exposure was reflected in three parasite within-host relationships: establishment (*ε*), mortality (*μ*) and fecundity (*F*) (Bishop and Stear, 1997). To quantify the degree of parasite exposure, and hence the acquisition of an immune response, the measure of *larvaldays* was devised. *Larvaldays* is a measure of the cumulative exposure to parasites, a function of the larval dose administered and the length of time the host experiences each individual larva, and was chosen to represent immune development due to its ability to account for the larval intake of one day to have effects on exposure in subsequent days, in addition to further incoming larvae (Eq. (1)). *Larvaldays* does not take into account larvae that have died or failed to establish, because the effect was found to be inconsequential, due to the relationship between *larvaldays* and the immune response (see below). All three affected responses (establishment, mortality and fecundity) were expressed as functions of *larvaldays*.

(1) $$Larvaldays=Larvaldays_{t-1}+\sum^{\text{⁣}}LI$$

where ∑ *LI* is the cumulative larval intake and *t* is time in days

##### 2.1.2.2 Defining and parameterising parasite burdens

In the absence of an immune response a maximum proportion of ingested larvae will establish; as the animal develops immunity, the proportion of the larvae that establish will decline until a plateau is reached (Klesius, 1988). A proportion of the established adult worms will die on any given day: in the absence of immunity a minimum mortality rate applies and as immunity develops this increases towards a maximum (Kao et al., 2000). Available data that measures the worm burden of parasitised calves for given larval challenges reflects the combination of the above two processes. These data alone cannot be used to show the separate effects of establishment and mortality.

Initially, the combined effect of establishment and mortality was plotted against *larvaldays* from the experiment A of Michel (1969), one of the very few experiments with such data. The data suggested an exponential relationship between *larvaldays* and the combined effect of establishment and mortality (*EM*), taking the form:

(2) $$EM=(EM_{max}-EM_{min})\times \exp\left(-k_{EM}\times larvaldays\right)+EM_{min}\;\;\;\;\;\;\;\;\;\;\;\;\;\;\;\;\;\;\;\;\;\;\;\;\;\;\;\;\;\;\;\;\;\;\;\;\;\;\;\;\;\;\;\;\;\;\;\;\;\;\;\;\;\;\;(\text{change in adult worm numbers}/day)$$

where *EM*_max_ is the maximum of combined establishment and mortality, *EM*_min_ is the minimum of combined establishment and mortality and *k*_EM_ is the constant relationship between *larvaldays* and the combined establishment and mortality level. The parameter values obtained from fitting the Eq. (2) to data were 0.82 (*EM*_max_), 0.08 (*EM*_min_) and 2.6E-08 (*k_EM_*) (R = 0.738, RMSE = 0.119). However, it was necessary to separate the effects of establishment and mortality in order to capture worm burden dynamics. It was, therefore, assumed that worm mortality rate followed the same sigmoidal pattern as described by Louie et al. (2005):

(3) $$\mu =\frac{(\mu_{max}-\mu_{min})\times \;{\left(Larvaldays\right)}^{2}}{k_{\mu }{}^{2}+{\left(Larvaldays\right)}^{2}}+\mu_{min}\;\;\;\;\;\;\;\;\;\;\;\;\;\;\;\;\;\;\;\;\;\;\;\;\;\;\;\;\;\;\;\;\;\;\;\;\;\;\;\;\;\;\;\;\;\;\;\;\;\;\;\text{(proportion adult worms/day)}$$

where *μ*_max_ is the maximum mortality, *μ*_min_ is the minimum mortality and *k_μ_* is a constant of the relationship between *larvaldays* and the mortality. The parameters were estimated using the values of Vagenas et al. (2007a) as a baseline, and adjusted to produce similar patterns of worm burden to those observed by Michel (1969). Values were estimated at 0.12 (*μ*_max_), 0.01 (*μ*_min_) and 4E + 06 (*k_μ_*). The remaining effect on the adult worm numbers after accounting for mortality was assumed to be attributable to the establishment rate (*ε*):

(4) $$\varepsilon =\frac{EM}{1-\mu }\;\;\;\;\;\;\;\;\;\;\;\;\;\;\;\;\;\;\;\;\;\;\;\;\;\;(\text{proportion larvae establishing}/day)$$

The modelled worm burdens were fitted to experimental data from experiment A of Michel (1969) to estimate establishment and mortality rate parameters within a dynamic system.

The likely stochastic nature of the pre-patent period was assumed to be normally distributed across this time period (mean = 21 days, SD = 1.64 days), and was estimated at whole day increments. This allowed for the gradual appearance of a worm burden rather than the otherwise sudden maturation of all larvae on a single day and can be represented as follows:

(5) $$MatureL_{x}=Larvae_{16}\times P_{x}$$

where *MatureL_x_* is the number of larvae maturing on day × from a given larval cohort, *Larvae*_16_ is the total number of larvae that will mature into adult worms from each larval cohort (administered 16 days previously) and *P*_x_ is the normal probability density function integrated over 1 day (and assumed to be negligible for t<17 and t> 25).

The worm burden could then be defined at time *t* as a function of the previous day’s worm burden and the newly matured adult worms (summed across all larval cohorts):

(6) $$WB_{t}=\left(1-\mu \right)\times WB_{t-1}+\underset{t}{\sum }MatureL_{x}$$

where *WB*_t_ is the new worm burden, *WB*_*t*−1_ is the previous days worm burden, μ is the parasite mortality and ∑ *MatureL_x_* is the sum of newly matured adult worms across all larval cohorts.

##### 2.1.2.3 Defining and parameterising worm fecundity and worm mass

As with parasite establishment, the fecundity (eggs/female) was assumed to decline towards a plateau as immunity was acquired (Michel, 1969). The immune response effect on fecundity was assumed to develop at a different rate to the establishment and mortality due to different underlying immune mechanisms (Stear et al., 1995, Prada Jiménez de Cisneros et al., 2014). As with *EM* the eggs per female was plotted against *larvaldays* from the experiment A of Michel (1969); the data suggest an exponential relationship between *larvaldays* and fecundity (*F*), taking the form:

(7) $$F=(F_{max}-F_{min})\times \exp\left(-k_{F}\times larvaldays^{}\right)+F_{min}\;\;\;\;\;\;\;\;\;\;\;\;\;\;\;\;\;\;\;\;\;\;\;\;\;\;\;\;\;\;\;\;\;\;\;\;\;\;\;\;\;\;(Eggs/female/day)$$

where *F*_max_ is the maximum number of eggs per female worm, *F*_min_ is the minimum number of eggs per female worm and *k_F_* is the constant of the relationship between *larvaldays* and fecundity. After fitting the equation to the data of Michel (1969) parameter values of 39 (*F*_max_), 6 (*F*_min_) and 2.9E-07 (*k*_F_) were obtained (R = 0.673, RMSE = 4.781). Key assumptions made were that the proportion of female worms was 0.55 (Verschave et al., 2014) and eggs develop at the same rate, irrespective of the age and length of the worm.

Worm mass was calculated to provide a more complete measure of parasite infection (Michel et al., 1978; Bishop and Stear, 1997); this accounted for worm length as affected by the density dependence effect, whereby worm size (and fecundity) decrease with increasing worm numbers (Michel et al., 1978). Worm length has been found to display strong positive correlation to adult worm fecundity (Stear and Bishop, 1999). The density dependence effect on worm mass was described according to Vagenas et al. (2007a) (Eqs. (8) and (9)):

(8) $$F_{Scaled}=F\times \left(\frac{WB}{WB_{Av}}\right){}^{DD}\;\;\;\;\;\;\;\;\;\;\;\;\;\;\;\;\;\;\;\;\;\;\;\;\;\;\;\;\;\;\;\;\;\;\;\;\;\;\;\;\;\;\;\;\;\;\;\;\;\;\;\;\;\;\;\;\;\;\;\;\;\;\;\text{(Eggs/female/day)}$$

where *WB_Av_* is the worm burden at which *F_Scaled_* is equal to *F* and provides an estimate at which intraspecific competition between worms occurs for limited resources, this was taken to be 15,000 adult worms per calf (Michel, 1969); and *DD* is a constant density dependence factor (−0.5).

Given the strong positive correlation between worm length and fecundity (Stear and Bishop, 1999), worm mass (*WM*) was calculated as:

(9) $$WM=WB\times F_{Scaled}$$

FECs (eggs/g faeces) were calculated as the total daily egg output divided by the daily faecal output as estimated from the passage of undigested dry matter (DM). The random nature of sampling FEC was modelled as a Poisson distribution (Torgerson et al., 2012), after taking into account the limit of detection of the modified McMaster technique to measure 25 eggs/g of faeces (Borgsteede and Hendriks, 1979, Geldhof et al., 2002). Grazing beef calves average a faecal DM content of 140–350 g DM/kg faeces (Allen et al., 1970, Young and Anderson, 1981, Bellosa et al., 2011, Jalali et al., 2015), hence it was assumed that faecal DM comprised 0.25 of the faecal matter.

##### 2.1.2.4 Parasite-induced anorexia

A reduction in voluntary feed intake accompanies parasitic infections (Kyriazakis et al., 1998, Kyriazakis, 2014). In *O. ostertagi* infection anorexia does not appear on average before 21 days post-infection (Szyszka and Kyriazakis, 2013), which coincides with the first appearance of adult worms. Anorexia was modelled as a direct function of the rate of acquisition of immunity as per Laurenson et al. (2011). The anorexia was then applied to actual feed intake, as described below, through a reduction parameter (RED). This was calculated as a direct function of the rates of firstly the combined effect of establishment and mortality and secondly of fecundity. Due to the differing physical units of the two immune measurements it was necessary to include a scaling factor; the rate of change in each response was scaled by the maximum possible change in the immune rate as follows:

(10) $$RED=C_{1}\left(\frac{dEM/dt}{EM_{max}-EM_{min}}+\frac{dF/dt}{F_{max}-F_{min}}\right)$$

where *C*_1_ is the scaling parameter, *DEM*/*dt* is the rate of change in combined establishment and mortality and *dF*/*dt* is the rate of change in fecundity.

A maximum RED for subclinical infections was considered (0.20 (Sandberg et al., 2006)). During the course of an infection RED will start at zero, rise to a maximum and then decline towards zero as immunity is acquired, however due to the slow development of immunity complete recovery may not occur over the time period considered. The reduction is considered a function of the desired feed intake to fulfil all requirements

(11) $$FI_{anorexic}=\left(1-RED\right)\times FI_{desired}\;\;\;\;\;\;\;\;\;\;\;\;\;\;\;\;\;\;\;\;\;\;\;\;\;\;\;\;\;\;\;\;\;\;\;\;\;\;\;\;\;\;\;\;\;\;\;\;\;\text{(kg/day)}$$

where *FI_anorexic_* is the feed intake of an anorexic calf and *FI_desired_* is the desired feed intake of the calf to fulfil all resource requirements.

##### 2.1.2.5 Protein loss

One of the consequences of *O. ostertagi* infection is damage to the abomasal tissue of the host, resulting in protein loss (Fox, 1993, Holmes, 1993). The protein loss is a function of both larval burden and worm mass (Parkins and Holmes, 1989, Scott et al., 2011); the general trend observed for both is a sigmoidal increase up to an asymptote as the mass increases (Vagenas et al., 2007a). The simplest equation to describe this was proposed to be a logistic equation with the rate values that have been determined heuristically to fit bodyweight losses in literature (Szyszka and Kyriazakis, 2013). Equations for the potential protein losses were represented as:

(12) $$PLM_{Pot}=\frac{Ploss_{max}\times Ploss_{target}\times \text{exp}\left(rLB\times LB\right)}{Ploss_{max}+Ploss_{target}\times \left(\text{exp}\left(rLB\times LB\right)-1\right)}\;\;\;\;\;\;\;\;\;\;\;\;\;\;\;\;\;\;\;\;\;\;\;\;\;\;\;\;\;\;\;\;\;\;\;\;\;\;\;\;\;\;\;\;\;\;\;\;\;\;\;\;\;\;\;\;\;\;\;\;\;\;\;\;\;(kg/day)$$

(13) $$PWM_{Pot}=\frac{Ploss_{max}\times Ploss_{target}\times \text{exp}\left(rWM\times WM\right)}{Ploss_{max}+Ploss_{target}\times \left(\text{exp}\left(rWM\times WM\right)-1\right)}\;\;\;\;\;\;\;\;\;\;\;\;\;\;\;\;\;\;\;\;\;\;\;\;\;\;\;\;\;\;(kg/day)$$

where *Ploss*_target_ is the target protein loss (0.0001 (Vagenas et al., 2007a, Laurenson et al., 2011)), *Ploss*_max_ is the maximum protein loss (0.5 kg/d, see Eq. (13)), *rLB* (8.5E-5) and *rWM* (8.0E-6) are the rates of protein loss associated with larval burden (*LB*) and worm mass (*WM*) respectively.

The total protein loss is considered as the sum of the protein loss caused by both larval burden and by worm mass (see Supplementary Data S1, Eqs. A.20 & A.21), up to a capped maximum protein loss. The maximum protein loss caused by parasitic burden is the maximum protein loss the host can withstand; if this is sustained across time calf mortality may eventually occur. As far as we are aware measurements of maximum protein loss for infected calves do not appear in the literature but have been reported for sheep, estimated as 0.01 kg/day (Laurenson et al., 2011). An allometric scaling parameter linking mature weight of sheep and cattle was used to scale the maximum protein loss for lambs to give a maximum value of 0.5 kg/d in calves.

(14) $$Ploss_{Max\left(Steer\right)}={\left(\frac{BW_{M\left(Steer\right)}}{BW_{M\left(Sheep\right)}}\right)}^{0.73}\times Ploss_{Max\left(Sheep\right)}\;\;\;\;\;\;\;\;\;\;\;\;\;\;\;\;\;\;\;\;\;\;\;\;\;\;\;\;\;\;\;\;\;\;\;\;\;\;\;\;\;\;\;\;\;\;\;\;\;\;\;\;\;\;\;\;\;\;\;\;\;(kg/day)\;$$

where *BW_M_*_(*Stee*r)_ is the mature weight of as teer, *BW_M_* is the mature body weight of a sheep, *Ploss_Max_*_(*Steer*)_ is the maximum calf protein loss and *Ploss*_*Max*(*Sheep*)_ is the maximum protein loss in lambs.

##### 2.1.2.6 Partitioning limited protein resources

Parasitised calves were assumed to have two additional functions to which they must allocate resources; damage repair and an immune response. As with healthy calves the maintenance requirements, along with damage repair were satisfied first (Coop and Kyriazakis, 1999). If these needs are not met then protein stores would be catabolised and eventually the calf would succumb to the consequences of the infection. Conversely, if nutrients remain after allocation to maintenance, they would be allocated between the two remaining functions of immunity and growth in proportion to their requirements (Coop and Kyriazakis, 1999). This allocation strategy is consistent with evidence of both reduced growth and immune development in nutritionally limited calves (Mansour et al., 1991, Mansour et al., 1992). Proportional allocation may allow the host to tolerate a small number of parasites providing opportunity for parasite recognition to develop over time, and hence prevent a large infection arising (Viney et al., 2005). The resource requirements for maintenance and growth are given in Section 1.1.3 of Supplementary Data S1, whereas the requirements for damage repair and the immune response were calculated as per Laurenson et al. (2011).

Due to protein allocation to the immune response there will be a reduction in protein loss caused by the parasites per se. The protein loss is then re-estimated following the reduction in worm mass and the spared protein added back to the available protein. The allocation to growth was estimated as:

(15) $$PAC_{Growth}=P_{Avail}-\left(PAC_{Imm}+PLoss\right)\text{(kg/day)}$$

where *PAC_Growth_* is the actual protein allocated to growth, *PAC_Imm_* is the protein allocated to immunity, *PLoss* is the protein loss after taking into account immunity and *P_Avail_* is the protein available to allocate to these processes.

#### 2.1.3 Investigating model behaviour

The model was used to investigate predictions for a range of parasite infection intensities. The default values for the model were Limousin x Holstein-Friesian steers allowed ad-libitum access to high quality grass (AFRC, 1993) for one grazing season (6–7 months from turnout). The default calf genotype was characterised according to EBLEX (2005) (Supplementary Data S1) with 106 kg of protein at maturity (*P_M_*), 207 kg of lipid at maturity (*L_M_*) and 0.0071 per day growth rate (*B*).

Model outputs were simulated for two challenge situations: the first tested the effect of different trickle doses of infective larvae administered daily. These were 3500, 7000 and 14,000 L_3_/d representing a range of larval intakes that might lead to subclinical infections (Szyszka and Kyriazakis, 2013). The second investigated the effect of weekly as opposed to daily trickle infections, to match the common experimental protocol for parasite administration (Wiggin and Gibbs, 1989, Xiao and Gibbs, 1992, Szyszka and Kyriazakis, 2013). The number of infective larvae administered for this purpose was a total of 210,000 L_3_ administered within a three week period. This was given either as a single dose, 3 doses of 70,000 L_3_ per week or as 21 doses of 10,000 L_3_/d. The daily outputs predicted by the model were worm burden, calf total egg output, FEC, feed intake and bodyweight.

### 2.2 Model sensitivity

In order to determine which parameters have the most significant effect on the model outputs a sensitivity analysis was conducted. An ANOVA was performed to determine the contribution of selected model parameters to variance of each output measure (Saltelli et al., 2010; Campolongo et al., 2011). The parameters selected were those for which the least confidence in actual values existed, but which appeared mechanistically important for model behaviour; this included 5 categories with a total of 12 parameters between them.

The following five categories were targeted for investigation:

1. Larval establishment and adult worm mortality as defined by 3 parameters: ***EM*_max_** – maximum proportion of larvae establishing and surviving as adult worms; ***EM*_min_** – minimum proportion of larvae establishing and surviving as adult worms; ***k_EM_*** – the constant relationship between *larvaldays* and surviving adult worms as affected by establishment and mortality.
2. Adult worm mortality as defined by 3 parameters:***μ***_**max**_ – maximum effect of mortality on adult worms; ***μ*_min_** – minimum effect of mortality on adult worms; ***k_μ_*** – the constant relationship between *larvaldays* and adult worm mortality.
3. The fecundity of female adult worms defined by 3 parameters: ***F*_max_**, – maximum number of eggs per female worm; ***F*_min_** – minimum number of eggs per female worm; ***k_F_*** – the constant relationship between *larvaldays* and number of female worms.
4. The rate of reduction in feed intake dependent on rate of immune acquisition: **C_1_**
5. The rate of protein loss, as defined by two rate parameters: ***rWM*** – the rate of protein loss associated with adult worm mass and ***rLB*** – the rate of protein loss associated with larval burden.

It was assumed that each parameter was normally distributed (Vagenas et al., 2007c), using the best-estimate value as the parameter mean and assuming a coefficient of variation of 20%. The possible values for the constant relationships with *larvaldays* levels (k) of establishment, mortality and fecundity were considered to follow a log-normal distribution in order to take into account the possible variation of a rate parameter over orders of magnitude. For the same reason, the likely rates of protein loss were also assumed to follow a log-normal distribution. The distributions of parameter values were divided into 5 sections, each section assumed to be of equal probability, and the mid-point value selected. This allowed for a simpler and more consistent comparison in the analysis by selecting 5 possible values for each of the 12 parameters and then generating random combinations of these values. Using Latin hypercube sampling (LHS), parameters were sampled without replacement for each section to give 5 sets of parameter combinations. This was repeated 50 times to give a total of 250 parameter combinations; this was considered a sufficient number of combinations to allow a 12-way ANOVA due to the large number of parameters that may affect each output. Each of the 250 combinations was then modelled over a 200 day period for the three separate challenge levels of 3500, 7000 and 14,000 L_3_/d and a record was taken of relevant outputs simulated. Each output set was then compared to the “best-estimate” output values (produced by the initial “best-estimate” parameters).

An ANOVA of constrained (Type III) sum of squares was conducted to analyse five defined outputs, viz. peak worm burden, time of peak worm burden, the peak total egg count, the peak reduction in feed intake and finally the final bodyweight. Significance was tested at the 99% level (p < 0.01) in all cases. A multiple linear regression was then conducted to determine the percentage change in outputs with respect to changes in parameter values. All model simulations and statistical analyses (ANOVA) were programmed in MATLAB (2012).

### 2.3 Model validation

The model was parameterised using data from experiment A of Michel (1969) due to its utility. To validate the model, graphical comparisons and statistical analyses were made on independent data from sets of published experiments. Model performance was assessed in terms of goodness-of-fit of the observed against predicted values for three selected outputs on a daily basis: adult worm burdens, total egg counts and FECs (Symeou et al., 2014). The literature studies selected for evaluation were based on the following criteria: (1) Infections were only with *O. ostertagi* and no other species were involved; (2) calves were infected during the growth phase; (3) calves were allowed access to ad-libitum, high quality feed; (4) calves were parasite naïve, i.e. had no prior experience of parasites before the experiment; (5) larval doses were administered either weekly or more frequently.

Only eleven studies met the above criteria and were used to test for the effects of different trickle doses on (1) worm burdens (Michel, 1969, Michel and Sinclair, 1969 experiment B; Michel, 1970); (2) total egg counts (Michel, 1969, Michel and Sinclair, 1969 experiment B); (3) FECs (Wiggin and Gibbs, 1989, Xiao and Gibbs, 1992, Mansour et al., 1992, Satrija and Nansen, 1993, Hilderson et al., 1995, Hilderson et al., 1993, Claerebout et al., 1996, Forbes et al., 2009). The experimental larval challenges were used as inputs to the model. It was assumed that there has been little to no selection for resistance to *O. ostertagi* and hence the parasitological parameters that can be seen as host specific, have remained unchanged over the time period considered by all experimental studies (Prakash, 2009). In order to compare the model outputs to observed FECs the former must be considered as eggs per gram of wet faecal matter, however the DM content will vary dependent on the feed. For all studies where feed type was specified, calves were fed corn silage, hay or concentrates which lead to a higher faecal DM content than when fed on grass (Young and Anderson, 1981; Van Bruchem et al., 1991); in these case the faecal DM content was assumed to be 350 g/kg DM.

The statistical analyses conducted to assess the goodness of fit for the purpose of model evaluation were as follows: (1) the correlation coefficients (R) were used to assess whether the simulated outputs followed the same pattern as observed values, with a value of unity signifying a perfect fit. (2) The coefficient of variation for the root mean square error (CV-RMSE) measured the closeness of observed and predicted values; a lower value signifies a closer match. (3) The relative error (E) determined the bias of predicted results, which is the total difference between predictions and observations. This revealed whether the results have been consistently over or under estimated in relation to the observed data; a positive E value indicates over estimation and a negative E value under estimation (Symeou et al., 2014).

$$E=\frac{\Sigma \frac{\left(O_{i}-P_{i}\right)}{O_{i}}}{n-1}$$

where *E* is the relative error, *O*_i_ is the observed value, *P_i_* is the predicted value and n is the total number of observations made.

The statistical significance of CV-RMSE was assessed by CV-RMSE_95%_, a value greater than this suggests that the predicted values are not within the 95% confidence intervals of the observed data (Symeou et al., 2014). The statistical significance of E was also tested with E_95%_, again an E value below this signifies predicted values fell within the 95% confidence intervals for the observed measurements (Symeou et al., 2014). Due to the nature of experimental infections conducted on cattle it was difficult to find an appreciable number of studies giving values taken from multiple calves at repeated time points. Thus for a subset of studies, it was possible to estimate the 95% confidence intervals on the experimental data (to compare with model deviation as measured by CV_RMSE and E).

## 3 Results

### 3.1 Model exploration

The model predictions on the effects of different trickle infectious doses are detailed below; the same predictions for the effects of different modes of administration of the same infectious doses are shown in Supplementary Data S2.

#### 3.1.1 The consequences of different levels of infection

**Fig. 2:**
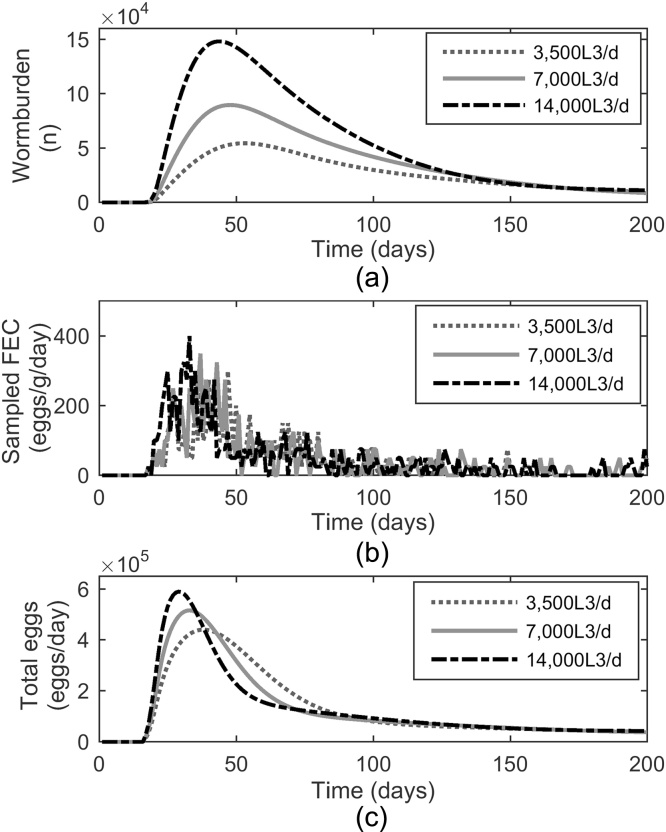
Predicted worm burdens (a), sampled daily faecal egg counts (FEC) (b) and daily faecal egg outputs (c) produced over time in calves administered one of 3 different infection doses of *Ostertagia ostertagi* L_3_ larvae: 3500, 7000 and 14,000 L_3_/day over a 200 day period. The FEC were subject to a random sampling error owing to external factors.

The worm burdens of a single calf infected with different trickle doses of *O. ostertagi* are shown in Fig. 2a. The rate of increase in worm burdens increased with increasing number of larvae administered, reaching a peak at 53, 48 and 44 days post infection (dpi) for the 3500, 7000 and 14,000 L_3_/d respectively. Worm numbers and their negative gradient of reduction started to decline faster at higher tickle doses. Worm burdens never reached zero even when immunity was developed in full. This is due to the assumption that a small number of larvae (8%) will continue to establish and from those a number will survive as adult worms (88%).

The FEC (eggs/g faeces) are a representation of the number of parasitic eggs found in a random sample of faeces (Fig. 2b). The distribution of eggs throughout the faeces is overdispersed and therefore the FEC had the potential to be largely over or under estimated, which is represented by the large day to day variation. A clear pattern in total egg numbers produced by all female worms per day in a calf is in Fig. 2c. The total egg counts show a similar pattern to worm burdens as this is reflective of the female worm populations, however the peak is slightly earlier at 33, 38 and 29 dpi for 3500, 7000 and 14,000 L_3_/d respectively. When comparing the relative maximum values of worm burdens and total egg outputs for different trickle doses, there was a greater difference across worm burdens. When compared to the low infection level of 3500 the peak worm burdens for 7000 and 14,000 L_3_/d were 1.65 and 2.72 times greater, whereas for the peak total egg counts the differences were not as pronounced, being 1.17 and 1.34 times greater respectively.

**Fig. 3:**
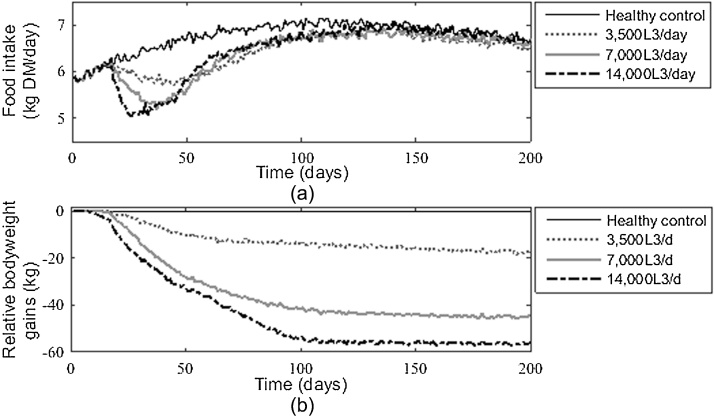
The predicted daily feed intake (a) and total relative bodyweight losses (in comparison to uninfected controls) (b) over time in calves administered 3 different infection levels of *Ostertagia ostertagi* L_3_ larvae: 3500, 7000 and 14,000 L_3_/day.

The feed intakes of calves given different trickle doses are shown in Fig. 3a, together with the intake of a healthy calf for comparison. A reduction in feed intake was observed for all infection levels; the extent of the reduction was greater for larger challenges. The point at which the maximum reduction in intake was observed was earlier for larger infection levels with recovery for 3500, 7000 and 14,000 L_3_/d starting at d 42, 37 and 25 pi respectively in reflection of the immune development. Feed intake returned to levels similar to those by the uninfected calf for the larger infection level by day 130; this was not the case for the lower levels of infection, where intake was slightly below to that of the uninfected calf.

The reductions in bodyweight of infected calves when compared to a healthy calf for different trickle doses are in Fig. 3b. The effect on bodyweight was greater with larger infection levels; this was predominantly due to reduced feed intake and the damage caused by worms. As the challenge level increased, disproportionate losses in weight gain were observed: a 152% increase in losses was observed from 3500 to 7000 L_3_/d compared to a 25% increase from 7000 to 14,000 L_3_/d. The maximum effects on the bodyweight appeared in the early stages of infection, where maximum bodyweight reductions of 3%, 9% and 12% were observed, for the three trickle doses respectively.

### 3.2 Model sensitivity

Table 1 shows the range of values for simulated outputs of the three traits: peak worm burden, time of peak worm burden (days) and final bodyweight (kg), when the selected model parameters were simultaneously varied. The numerical ranges of the outcomes of maximum worm burden were largest for higher challenge levels. The range for final bodyweights, however, was the same for all challenge levels. Parameters that had a significant effect are reported in order of magnitude of effect on the given output (i.e. the output is most sensitive to the first noted parameter). P values are given in Supplementary Table S1.

#### 3.2.1 Parasitism outputs

Worm burdens were significantly affected by 3 parameters: ***k_EM_*** (the constant relationship between *larvaldays* and its effect on establishment and mortality); ***EM*_max_** (maximum effect of establishment and mortality) and ***k_μ_*** (the constant relationship between *larvaldays* and mortality) when significance was fixed at the 99% significance level (p < 0.01). Time of peak worm burden was significantly affected by ***k_EM_***, ***EM*_max_** and ***μ*_max_** (maximum mortality) for all infection levels. The total egg counts were found to be sensitive to a large number of parameters with 4 having significant effect for all infection levels. Affecting parameters were ***k_EM_***; ***F*_max_** (maximum fecundity) and ***EM*_max_**. Additionally, total egg counts were significantly affected by ***μ*_max_** at 14,000 L_3_/d, whereas the effect was not significant for other infection levels.

**Fig. 4:**
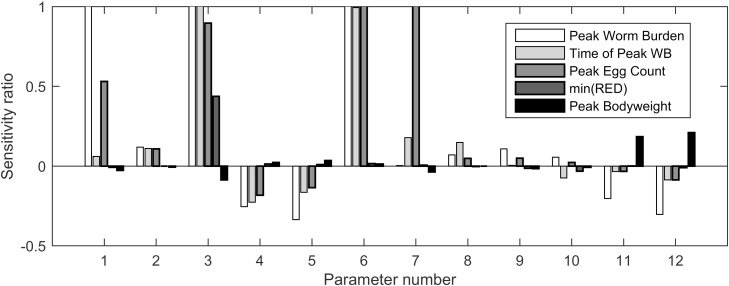
The sensitivity ratio of each of the 5 outputs considered (value and time of peak worm burden, peak faecal egg count, peak of reduction in feed intake and final bodyweight) in relation to each of the model parameters considered (1–12) when a calf was infected with 3500 L_3_/d. The parameters were firstly the immune parameters (1–9): the combined effect of establishment and mortality on adult worm burdens (maximum, minimum and rate): *EM*_max_ (1), *EM*_min_ (2), *k_EM_* (3); the effect of mortality of adult worms (maximum, minimum and rate): *μ*_max_ (4), *μ*_min_ (5), *k_μ_* (6); the fecundity (eggs) of female adult worms (maximum, minimum and rate): *F*_max_ (7), *F*_min_ (8), *k*_F_ (9). The performance parameters (9–12) considered were; the rate of reduction in feed intake dependent on rate of immune acquisition: *C*_1_ (10); the rate of protein loss caused by adult worms *rWM* (11) and by larvae *rLB* (12). The sensitivity analysis was conducted by the Latin hypercube sampling technique.

The relative effect of changing each parameter can be seen in the linear regression plots, as demonstrated for the infection level of 14,000 L_3_/d (Fig. 4). The sensitivity ratio plotted indicates the relative change in the output for a given relative change in the parameter; for example, a coefficient of 1 indicates that a 10% increase in the parameter produces a 10% increase in the particular model output. The largest infection level of 14,000 L_3_/d was chosen as this appeared to be the most sensitive to parameter changes. From these plots it was clearly seen that measures of parasitism were most sensitive to the constant relationship between *larvaldays* and the combined effect of establishment and mortality. Conversely, changes in the parasite-related parameters of ***EM*_min_** (minimum effect of establishment and mortality), ***μ*_min_** (minimum mortality), ***F*_min_** (minimum fecundity), ***k_F_*** (the constant relationship between *larvaldays* and mortality) and performance-related parameters **C*_1_*** (the rate of reduction in feed intake dependent on rate of immune acquisition), ***rWM*** (rate of protein loss associated with worm mass and ***rLB*** (rate of protein loss associated with larval burden) barely affected the outcomes.

#### 3.2.2 Performance outputs

The maximum reduction in feed intake was significantly impacted by ***C_1_*** (the rate of reduction in feed intake dependent on rate of immune acquisition) and ***k_EM_*** (the constant relationship between *larvaldays* and its effect on establishment and mortality). Bodyweights were significantly impacted by ***k_EM_***, ***rWM*** (rate of protein loss associated with worm mass), and ***rLB*** (rate of protein loss associated with larval burden) for all infection levels.

### 3.3 Model validation

The model was tested using published experimental studies, the statistical comparsions are displayed in Table 2. The graphical comparsions for the best and worst fits are shown; for worm burden the examples selected were Michel (1970) and Michel and Sinclair (1969); for total egg outputs Michel and Sinclair (1969) and for FECs Satrija and Nansen (1993) and Wiggin and Gibbs (1989). The remaining comparisons are presented in Supplementary Data S3.

**Fig. 5:**
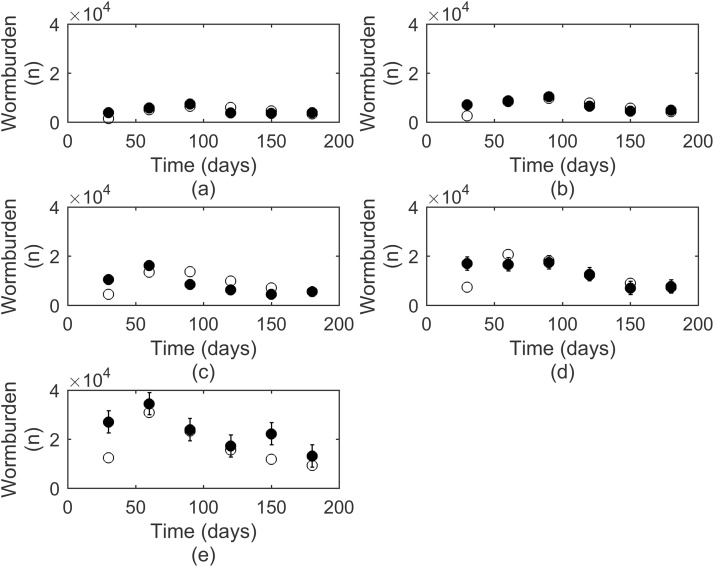
A comparison of the observations (●) by Michel (1970) to simulated predictions (○) for worm burdens produced by Ostertagia ostertagi infections of (a) 2 00 L_3_/d; (b) 340 L_3_/d; (c) 570 L_3_/d; (d) 950 L_3_/d; (e) 1600 L_3_/d. Each measurement was taken from 5 calves for each point.

**Fig. 6:**
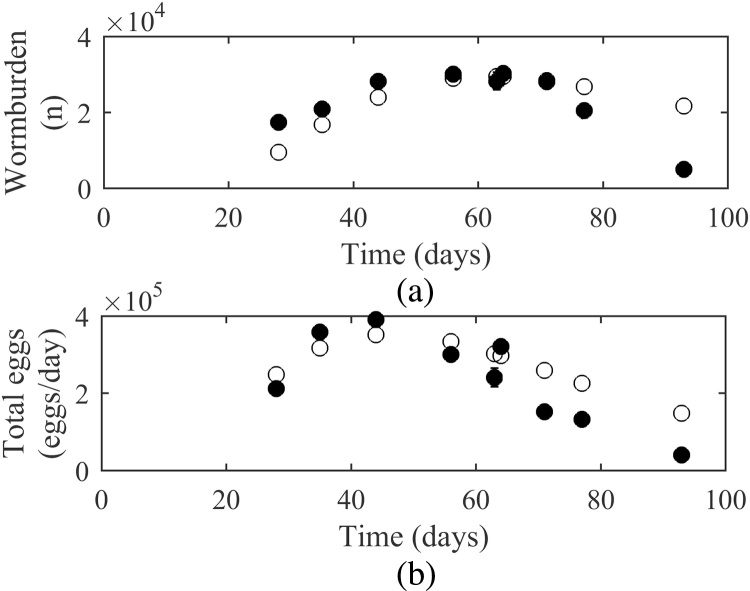
A comparison of experimental observations (●) by Michel and Sinclair (1969) to simulated predictions (○) for (a) worm burdens and (b) total eggs counts produced by an infection level of 1500 L_3_/d. Each point is based on measurements from one calf, with the exception of day 63 which is based on measurements from 2 calves.

In the majority of cases the comparsion between experimental and model observations showed a similar pattern for worm burdens with increasing worm burdens up to a peak followed by a decline; this was reflected in the high positive correlation coefficients between 0.581 and 0.834. A graphical comparison of model predictions and observations for Michel (1970) is presented in Fig. 5. Although the CV-RMSE did not fall within the 95% level, suggesting a large amount of dispersal from the observed results, the E value fell well within the E_95%_ suggesting there was no bias and predictions were not consistently over or under estimated compared to observed values. The exception to this pattern was Michel and Sinclair (1969) in which a faster decline in worm burdens was observed (Fig. 6a). This was reflected in the lower R value and larger negative E value, showing a consistent overestimation by the model.

Of the aforementioned studies meeting the validation criteria only two provided total egg outputs; similarly to the worm burdens the observations revealed total eggs reached a maximum early on in the infection and decreased from this point onwards. Model predictions were in reasonable agreement with the observed values for both experiment B of Michel (1969) (Supplementary Fig. S1) and Michel and Sinclair (1969). The latter showed a close correspondance with a high R correlation coefficient of 0.926; however as a consequence of the pattern of worm burden the E value showed again a consistent overestimation of results by the model (Fig. 6b).

**Fig. 7:**
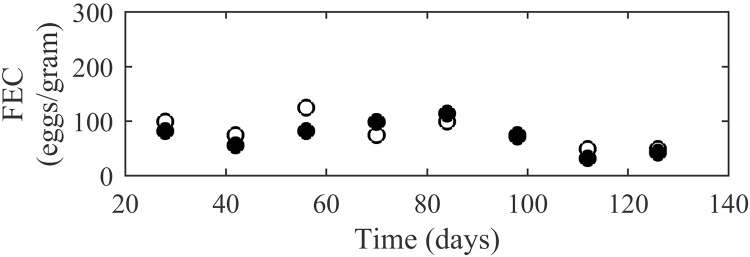
A comparison of experimental observations (●) by Satrija and Nansen (1993) to simulated predictions (○) for faecal egg outputs per gram of fresh faeces resulting from a weekly infection of 1250 larvae. Each measurement was taken for 6 calves.

**Fig. 8:**
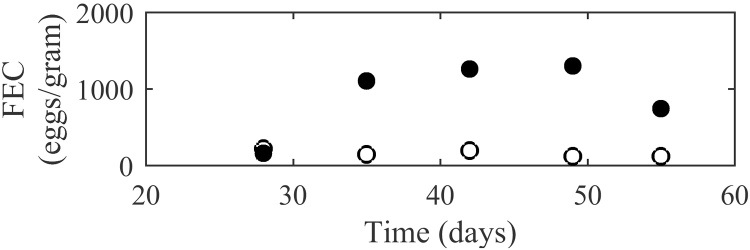
A comparison of experimental observations (●) by Wiggin and Gibbs (1989) to simulated predictions (○) for faecal egg outputs per gram of fresh faeces produced by a weekly infection of 30,000 larvae. Each measurement was taken for 12 calves.

In general the observed pattern of FECs was similar to that of total egg outputs: increasing to a peak early on in the infection and then consistently decreasing. The pattern was not as evident due to the sampling error incorporated for FEC counting; this was reflected in the R values given in Table 2. An example of a good fit was Satrija and Nansen (1993) in which a relatively low CV-RMSE and E value indicate a close fit between results and minimal bias, this is represented graphically in Fig. 7. However not all experiments provided such strong support to the model, in particular Wiggin and Gibbs (1989) for which FEC offered an extremely weak R coefficient of −0.059 suggesting the observed pattern was not well replicated by model predictions (Fig. 8). This was accompanied by an extremely large CV-RMSE value of 97.1 and a largely positive E value suggesting a gross underestimation by the model, which can clearly be seen in Fig. 8. However, it can be observed that the FEC values reported in Wiggin and Gibbs (1989) are noticeably larger than typical published values.

## 4 Discussion

The overall aim of this paper was to develop a model that accounted for the interactions between *O. ostertagi* parasitism and first season grazing calves, under UK conditions. Although the model was deterministic, it was constructed with the view of developing it into a stochastic one, to allow for the investigation of different methods of control of the parasite, including selection for host resistance (Laurenson et al., 2012a). Larval intake was considered an input to the model, but there are plans to account for parasite populations in the environment in the manner similar to Laurenson et al. (2012a).

Although there are a number of models focusing on predicting the epidemiology of *O. ostertagi* (Gettinby et al., 1979; Gettinby and Paton, 1981; Chaparro and Canziani, 2010), currently there are only two models that specifically aim to investigate within-host interactions between calf host and *O. ostertagi*. The PARABAN model (Smith and Grenfell, 1985; Smith et al., 1987; Grenfell et al., 1987a, Grenfell et al., 1987b) was specifically developed to account for the rate of change in parasite populations within hosts and the environment, and has been used to investigate the effectiveness of anthelmintic treatment on parasite dynamics. This model, however, does not account for the consequences of parasitism on host performance and its creators recognised its limitations in this respect (Smith, 1997).

Ward (2006a) attempted to account for the consequences of parasitism on calf performance by developing an animal growth model and by considering the effects of parasitism on host feed intake and metabolism. Parasite dynamics were expressed by the same equations that formed the basis of the above model (Smith et al., 1987). This implies that parasite establishment and fecundity were considered a function of time, as opposed to being a function of the development of the immune response (Smith and Grenfell, 1994); the only description of calf state used in the model was its bodyweight. A consequence of these assumptions would be an under- or over-estimation of calf performance during parasitism, as was indeed the case in the validation of the model by Ward (2006b). This could arise, for example, by over or under expression of the immune function to parasites as a consequence of nutrition (Ploeger et al., 1995, Coop and Kyriazakis, 1999).

The previously developed models identify the challenges associated with the development of a model that predicts the interactions between *O. ostertagi* and calves. In our model the animal state was characterised by calf degree of maturity (current protein mass divided by mature protein mass) and level of fatness, consistent with other animal growth models (Emmans and Kyriazakis, 2001), and by the cumulative exposure to parasitic challenge (*larvaldays*). The former feature enables simulation of different genotypes. A further attraction of describing the calf through these traits is that it is possible to introduce variation and co-variation in them and as a consequence to convert a deterministic model into a stochastic one (Vagenas et al., 2007c, Laurenson et al., 2012b). The consideration of *larvaldays* enabled to relate the immune response of the animal to be linked to the duration of parasite exposure, which is hypothesised to have greater effect on immune acquisition than the level of infection per se (Hilderson et al., 1993). Hence this model was able to portray differences in rate of immune development at different levels of infection.

Protein loss, which is the main consequence of gastrointestinal parasite challenge (Taylor et al., 1989), was related to current worm mass and larval burden, as opposed to worm burden and larval intake (Ward, 2006a). It was not possible to treat the impact of larvae mass similarly to worm mass, due to the difficulties in estimating the impact of immunity on larval mortality. On entering the host the model immediately discarded any larvae that failed to establish hence potentially resulting in an underestimation of the larval burden. Although there is currently little quantitative information about parasite-induced protein loss in calves, some assumptions were made, consistent with the quantitative estimates of protein loss during abomasal parasitism in sheep (Laurenson et al., 2011) and our current estimates of the effects of *O. ostertagi* on calf productivity (Szyszka and Kyriazakis, 2013). Better estimates of these relationships will enhance model accuracy.

The basis of the causal reduction in feed intake during parasitism has been the subject of considerable debate (Fox et al., 1989b, Kyriazakis et al., 1998, Laurenson et al., 2011). Feed intake reduction during parasitism was related to the rate of change in each of the immune parameters: this was in order to relate parasite-induced anorexia to the development of the immune response, as has been suggested by Sandberg et al. (2006) and Kyriazakis, 2011, Kyriazakis, 2014. The rapid recovery in feed intake post administration of anthelmintics in cattle (Bell et al., 1990) and other ruminants (Kyriazakis et al., 1996), suggests that anorexia is not a consequence of pathology, but is inextribaly linked to the stimulation of the immune response caused by the exposure to the parasites. Feed intake recovers when the immune reponse is fully developed (Kyriazakis et al., 1996, Sandberg et al., 2006); however it was assumed that there would be no compensatory increase in feed intake and perfomance (Kyriazakis and Houdijk, 2007). The existence of such compensatory response would affect the predictions of the model in terms of calf performance, but not its parasitological outputs.

The assumptions made about within host parasite populations and the interactions between host and parasite lead to a number of model behaviours. The rate of reduction in worm numbers was more rapid for higher infection pressures; this was a reflection of the model assumption that the development of immunity was dependent on the cumulative exposure to larvae. Worm burdens never reached zero even when immunity had developed in full, consistently with the idea of incomplete and slow development of immunity to *O. ostertagi* in relation to other parasite species (Klesius, 1988; Hilderson et al., 1993). We did not observe a relationship between infection pressure and the plateau of within host worm burden, as suggested by Cattadori et al. (2005); this was a reflection of the absence of an epidemiological component in our model.

Anorexia became evident around the same time for all infection levels; this was a result of a threshold level of immune acquisition achieved at a similar time for each challenge dose, consistent with Szyszka and Kyriazakis (2013). In addition it has been shown that feed intake is not affected during the stage of larval development (Michel, 1969; Fox et al., 1989a, Szyszka and Kyriazakis, 2013). Feed intake also began to recover earlier for higher infection levels. This was a reflection of the assumption for faster immune acquisition and a higher desired intake to meet increased nutrient demands; more heavily parasitized calves must have larger requirements for repair and immunity (Sandberg et al., 2006). The total duration of anorexia was shortest for larger infection levels with no clear recovery in feed intake occurring for the lower levels. This is consistent with Herlich (1980) who found that the duration of anorexia seemed to be unrelated to the size of worm burden across cattle age groups infected with *O. ostertagi,* with cattle of 24 months showing large worm burdens but without signs of anorexia. In contrast Herlich (1980) concluded that of the age groups considered (2, 4/5, 12 and 24 months) only the 2 month old calves appeared to show ‘resistance’ to parasitic infection, implying the highest development of immunity, and coincidentally the highest incidence of anorexia.

A sensitivity analysis was conducted to identify parameters of key influence; the LHS was chosen as this method attempts to cover the widest space of possible parameter combinations. As far as we are aware, this has been the first attempt to apply the methodology in the validation of parasitological models. The approach requires fewer simulations than the Monte Carlo method as it is guaranteed to cover more uniformly the complete range of possibilities. Conversely a Monte Carlo simulation, which selects values at random, may generate clusters of similar parameter combinations while failing to probe other important regions of the parameter space.

In order to place any confidence in the model it was necessary to validate it against published literature. Parasitological traits were validated by comparing observed and simulated outputs for worm burdens, total egg outputs and FEC. In general model predictions showed close correlations to observations for worm burdens and total egg outputs, although relatively large CM-RMSE values suggested high levels of individual variation. In most cases FECs also showed a good fit, although these were rather more variable as a result of disparities in patterns of feed intake and faecal consistency (Vagenas et al., 2007b). Experiments will always be restricted by the number of animals involved; simulations studies are not limited by this, but can take into account between animal variation. Of the relevant studies many were performed a number of years ago; since then calves have been selected for performance traits, but little to no selection for resistance appears to have taken place (Prakash, 2009). Owing to a lack of experimental studies investigating the effect of sub-clinical challenge levels on calf DM intake it was not possible to validate performance; inference was made from bodyweight losses comparatively to control animals. A general review of the literature on feed intake during *O. ostertagi* infection showed varied food intake patterns between studies (Fox et al., 1989a Szyszka and Kyriazakis, 2013). It has been observed that duration and magnitude of parasite-induced anorexia are both strain dependent with a variance of up to 8 days between strains (Herlich et al., 1984).

The limitations of the model predictions point towards the need to develop a population model, as opposed to a deterministic model to account for calf—*O. ostertagi* interactions. To account for discrepancies between studies and for variation within them resulting from calf genetic variation, a stochastic herd-based model needs to be developed. Vagenas et al. (2007c) and Laurenson et al. (2012b) have described the challenges associated with this task for the development of a simulation model that accounted for the interactions between sheep and *T. circumcincta*. Nevertheless, such a development is a necessary step to address the consequences of management on the parasitism of a population of calves, especially given the move towards the development of targeted selective treatments in order to reduce the rate of selection for anthelmintic resistance (Charlier et al., 2014) whereby individuals are treated only when a given trait crosses a threshold level.

## 5 Conclusions

A dynamic, deterministic model to account for the interactions between calves and *O. ostertagi* has been developed. Although the model was developed for a specific calf genotype given *ad libitum* access to high quality grass, the model is able to apply to other genotypes and be extended for different nutritional scenarios. Comparisons of model outputs to experimental observations highlighted both model strengths and weaknesses. Reliance of the model on expressing the development of the immune responses affecting parasite populations within the host, points towards the need to collect further data to define such relationships. In this respect the model has a heuristic value. A major strength of the model is its ability to be converted into a population model and hence be used as a tool to investigate the consequences of parasitism in a group of calves subjected to different management treatments.

## Figures and Tables

**Table 1:** The range of model outcomes for the three parasitological outputs of peak worm burden, timing of peak worm burden, and final bodyweight are shown for simulations of the model run at three challenge levels of 3500, 7000 and 14,000 L_3_/d. The simulations for each challenge level were run using parameter combinations generated using the Latin hypercube sampling method whereby combinations were randomly selected to best cover the area of possible outcomes. Each.

Larval Challenge (L_3_/day)	Peak worm burden	Time of peak worm burden (days)	Final Bodyweight (kg)
3500	0.146–2.06 × 10^5^	31–132	465–564
7000	0.241–4.15 × 10^5^	29–112	463–563
14,000	0.389–5.06 × 10^5^	27–96	463–563

**Table 2:** The outcomes of statistical analyses used to assess goodness-of-fit between predictions and observed and experimental results of worm burdens, total egg outputs and faecal egg counts. Values for the R correlation coefficient, the coefficient of variation of the root mean square error (CV RMSE) and the relative error (E) are all given to 3 significant figures. The 95% confidence interval of experimental data is estimated where possible; in some cases standard deviations were not provided as only one calf was used for each measurement.

Measurement output	Source	R	CV RMSE (%)	CV RMSE_95%CI_	E (%)	E_95%_
Worm burdens	Michel (1970)	0.834	39.2	36.1	3.58	24.3
Worm burdens	Michel (1969) Experiment B	0.728	43.0	N/A	−4.30	N/A
Total eggs		0.684	61.4	N/A	−16.7	N/A
Worm burdens	Michel and Sinclair (1969)	0.581	27.6	N/A	−28.9	N/A
Total eggs		0.926	28.4	N/A	−45.4	N/A
Faecal Egg Counts	Claerebout et al. (1996)	0.728	71.3	N/A	67.2	N/A
Faecal Egg Counts	Forbes et al. (2009)	0.671	56.6	N/A	48.7	N/A
Faecal Egg Counts	Hilderson et al. (1993)	0.368	80.5	N/A	−8.28	N/A
Faecal Egg Counts	Hilderson et al. (1995)	0.798	62.1	N/A	66.2	N/A
Faecal Egg Counts	Mansour et al. (1992)	0.654	35.9	N/A	−13.2	N/A
Faecal Egg Counts	Satrija and Nansen (1993)	0.699	29.1	N/A	−17.7	N/A
Faecal Egg Counts	Wiggin and Gibbs (1989)	−0.0590	97.1	N/A	65.0	N/A
Faecal Egg Counts	Xiao and Gibbs (1992)	0.813	64.8	N/A	65.2	N/A

N/A—not applicable.

## Appendix A Supplementary data

Fig. S1 Worm burden (a), daily faecal egg output (b), daily food intake (c) and total relative bodyweight loss (in comparison to uninfected controls, losses are cumulative over time) (d) incurred over time in calves given a total of 210,000 *Ostertagia ostertagi* larvae over three weeks administered either daily (10,000 per day trickle challenge), as three weekly doses of 70,000, or as a single dose at the start of the period.

Fig. S2 A comparison of experimental observations (●) by Michel (1969) experiment B to simulated predictions (○) for worm burdens resulting from infection doses of (a) 500 larvae per day; (b) 1000 larvae per day; (c) 1500 larvae per day and total eggs per day resulting from infection levels of (d) 500 larvae per day; (e) 1000 larvae per day; (f) 1500 larvae per day. Each experimental data point is based on measurements from a single calf.

Fig. S3 A comparison of experimental observations (●) by Claerebout et al. (1996) to simulated predictions (○) for faecal egg outputs per gram of fresh faeces produced by an infection level of 20,000 larvae per week, administered in 3 doses, for 21 weeks. Each measurement was taken for 6 calves.

Fig. S4 A comparison of experimental observations (●) by Forbes et al. (2009) to simulated predictions (○) for faecal egg outputs per gram of fresh faeces produced by an infection level of 70,000 larvae per week, administered in 3 doses, for 8 weeks. Each measurement was taken for 5 calves.

Fig. S5 A comparison of experimental observations (●) by Hilderson et al. (1993) to simulated predictions (○) for faecal egg outputs per gram of fresh faeces produced by infection levels of (a) 5000 larvae per week; (b) 10,000 larvae per week; (c) 20,000 larvae per week; (d) 40,000 larvae per week, all administered in 3 doses a week for 17 weeks. Each measurement was taken for 4 calves.

Fig. S6 A comparison of experimental observations (●) by Hilderson et al. (1995) to simulated predictions (○) for faecal egg outputs per gram of fresh faeces produced an infection level of 20,000 larvae per week, administered in 3 doses, for 17 weeks. Each measurement was taken for 5 calves.

Fig. S7 A comparison of experimental observations (●) by Mansour et al. (1992) to simulated predictions (○) for faecal egg outputs per gram of fresh faeces produced by an infection level of 3000 larvae administered every other day for 6 weeks. Each measurement was taken for 6 calves.

Fig. S8 A comparison of experimental observations (●) by Xiao and Gibbs (1992) to simulated predictions (○) for faecal egg outputs per gram of fresh faeces produced by a weekly infection of 10,000 larvae for 14 weeks. Each measurement was taken for 5 calves.

## References

[bib0005] AFRC (1993). Energy and Protein Requirements of Ruminants: An Advisory Manual Prepared by the AFRC Technical Committee on Responses to Nutrients.

[bib0010] Allen W., Sweasey D., Berret S., Nancy Hebert C., Patterson D. (1970). Clinical pathology of ostertagiasis in calves during prolonged experimental infection. J. Comp. Pathol..

[bib0015] Bell S.L., Thomas R.J., Ferber M.T. (1990). Appetite, digestive efficiency, feed utilization and carcass evaluation of housed calves naturally infected with gastrointestinal nematodes. Vet. Parasitol..

[bib0020] Bellosa M.L., Nydam D.V., Liotta J.L., Zambriski J.A., Linden T.C., Bowman D.D. (2011). A comparison of fecal percent dry matter and number of Cryptosporidium parvum oocysts shed to observational fecal consistency scoring in dairy calves. J. Parisitol..

[bib0025] Bishop S.C., Stear M.J. (1997). Modelling responses to selection for resistance to gastro-intestinal parasites in sheep. Anim. Sci..

[bib0030] Borgsteede F.H., Hendriks J. (1979). Experimental infections with *Cooperia oncophora* (Railliet, 1918) in calves: results of single infections with two graded dose levels of larvae. Parasitology.

[bib0035] Campolongo F., Saltelli A., Cariboni J. (2011). From screening to quantitative sensitivity analysis: a unified approach. Comput. Phys. Commun..

[bib0040] Cattadori I.M., Boag B., Bjørnstad O.N., Cornell S.J., Hudson P.J. (2005). Peak shift and epidemiology in a seasonal host-nematode system. Proc. R. Soc. B.

[bib0045] Chaparro M.A.E., Canziani G.A. (2010). A discrete model for estimating the development time from egg to infecting larva of *Ostertagia ostertagi* parametrized using a fuzzy rule-based system. Ecol. Modell..

[bib0050] Charlier J., Morgan E.R., Rinaldi L., Dijk J., Van Demeler J., Höglund J., Hertzberg H., Ranst B., Van Hendrickx G., Vercruysse J., Kenyon F. (2014). Review practices to optimise gastrointestinal nematode control on sheep, goat and cattle farms in Europe using targeted (selective) treatments. Vet. Rec..

[bib0055] Claerebout E., Hilderson H., Meeus P., De Marez T., Behnke J., Huntley J., Vercruysse J. (1996). The effect of truncated infections with *Ostertagia ostertagi* on the development of acquired resistance in calves. Vet. Parasitol..

[bib0060] Coop R.L., Kyriazakis I. (1999). Nutrition-parasite interaction. Vet. Parasitol..

[bib0065] Doeschl-Wilson A., Vagenas D., Kyriazakis I., Bishop S. (2008). Exploring the assumptions underlying genetic variation in host nematode resistance. Genet. Sel..

[bib0070] EBLEX, 2005. http://www.eblex.org.uk/wp/wp-content/uploads/2013/06/beefactionforprofit1betterreturnsfromcontinentalcrosssteers.pdf (accessed: June 2015).

[bib0075] Edmonds M.D., Johnson E.G., Edmonds J.D. (2010). Anthelmintic resistance of *Ostertagia ostertagi* and *Cooperia oncophora* to macrocyclic lactones in cattle from the western United States. Vet. Parasitol..

[bib0080] Emmans G.C., Kyriazakis I. (1997). Models of pig growth: problems and proposed solutions. Livest. Prod. Sci..

[bib0085] Emmans G.C., Kyriazakis I. (2001). Consequences of genetic change in farm animals on food intake and feeding behaviour. Proc. Nutr. Soc..

[bib0090] Emmans G.C. (1997). A method to predict the food intake of domestic animals from birth to maturity as a function of time. J. Theor. Biol..

[bib0095] Forbes A.B., Warren M., Upjohn M., Jackson B., Jones J., Charlier J., Fox M.T. (2009). Associations between blood gastrin ghrelin, leptin, pepsinogen and *Ostertagia ostertagi* antibody concentrations and voluntary feed intake in calves exposed to a trickle infection with *O. ostertagi*. Vet. Parasitol..

[bib0100] Fox M.T., Gerrelli D., Pitt S.R., Jacobs D.E., Gill M., Gale D.L. (1989). Ostertagia ostertagi infection in the calf: effects of a trickle challenge on appetite, digestibility, rate of passage of digesta and liveweight gain. Res. Vet. Sci..

[bib0105] Fox M.T., Gerrelli D., Pitt S.R. (1989). Ostertagia ostertagi infection in the calf: effects of a trickle challenge on the hormonal control of digestive and metabolic function. Res. Vet. Sci..

[bib0110] Fox M.T. (1993). Pathophysiology of infection with *Ostertagia ostertagi* in cattle. Vet. Parasitol..

[bib0115] Geldhof P., Claerebout E., Knox D., Vercauteren I., Looszova A., Vercruysse J. (2002). Vaccination of calves against Ostertagia ostertagi with cysteine proteinase enriched protein fractions. Parasite Immunol..

[bib0120] Gettinby G., Paton G. (1981). The role of temperature and other factors in predicting the pattern of bovine *ostertagia* spp. infective larvae on pasture. J. Therm. Biol..

[bib0125] Gettinby G., Bairden K., Armour J., Benitez-usher C. (1979). A prediction model for bovine ostertagiasis. Vet. Rec..

[bib0130] Grenfell B.T., Smith G., Anderson R.M. (1987). A mathematical model of the population biology of *Ostertagia osertagi* in calves and yearlings. Parasitology.

[bib0135] Grenfell B.T., Smith G., Anderson R.M. (1987). The regulation of *Ostertagia ostertagi* populations in calves: the effect of past and current experience of infection on proportional establishment and parasite survival. Parasitology.

[bib0140] Höglund J., Morrison D., a Charlier J., Dimander S.-O., Larsson A. (2009). Assessing the feasibility of targeted selective treatments for gastrointestinal nematodes in first-season grazing cattle based on mid-season daily weight gains. Vet. Parasitol..

[bib0145] Höglund J., Dahlström F., Sollenberg S., Hessle A. (2013). Weight gain-based targeted selective treatments (TST) of gastrointestinal nematodes in first-season grazing cattle. Vet. Parasitol..

[bib0150] Herlich H., Gasbarre L.C., Douvres F. (1984). Infectivity and Pathogenicity of Three Isolates of *Ostertagia ostertagi* in cattle. Vet. Parasitol..

[bib0155] Herlich H. (1980). Ostertagia ostertagi infection and reinfection in cattle of different ages. Am. J. Vet. Res..

[bib0160] Hilderson H., Vercruysse J., de Graaf D.C., Bastiaensen P., Fransen J., Berghen P. (1993). The presence of an early L4 larvae population in relation to the immune response of calves against *Ostertagia ostertagi*. Vet. Parasitol..

[bib0165] Hilderson H., Vercruysse J., Claerebout E., De Graaf D.C., Fransen J., Berghen F.P. (1995). Interactions between *Ostertagia ostertagi* and *Cooperia oncophora* in calves. Vet. Parasitol..

[bib0170] Holmes P.H. (1993). Interactions between parasites and animal nutrition: the veterinary consequences. Proc. Nutr. Soc..

[bib0175] Jalali A.R., Weisbjerg M.R., Nadeau E., Randby Å.T., Rustas B., Eknæs M., Nørgaard P. (2015). Effects of forage type, animal characteristics and feed intake on faecal particle size in goat sheep, llama and cattle. Anim. Feed Sci. Technol..

[bib0180] Kao R.R., Leathwick D.M., Roberts M.G., Sutherland I.A. (2000). Nematode parasites of sheep: a survey of epidemiological parameters and their application in a simple model. Parasitology.

[bib0185] Klesius P.H. (1988). Immunity to *Ostertagia ostertagi*. Vet. Parasitol..

[bib0190] Kyriazakis I., Emmans C. (1995). The voluntary feed intake of pigs given feed based on wheat bran, dried citrus pulp and grass meal, in relation to measurements of feed bulk. Br. J. Nutr..

[bib0195] Kyriazakis I., Houdijk J., Wiseman J., Varley J., McOrist M., Kemp B. (2007). Food intake and performance of pigs during health, disease and recovery. Paradigms in Pig Science.

[bib0200] Kyriazakis I., Anderson D.H., Coop R.L., Jackson F. (1996). The pathophysiology and development of immunity during long-term subclinical infection with *Trichostrongylus colubriformis* of sheep receiving different nutritional treatments. Vet. Parasitol..

[bib0205] Kyriazakis I., Tolkamp B., Hutchings M. (1998). Towards a functional explanation for the occurrence of anorexia during parasitic infections. Anim. Behav..

[bib0210] Kyriazakis I. (2011). Opportunities to improve nutrient efficiency in pigs and poultry through breeding. Animal.

[bib0215] Kyriazakis I. (2014). Pathogen-induced anorexia: a herbivore strategy or an unavoidable consequence of infection?. Anim. Prod. Sci..

[bib0220] Laurenson Y.C.S.M., Bishop S.C., Kyriazakis I. (2011). In silico exploration of the mechanisms that underlie parasite-induced anorexia in sheep. Br. J. Nutr..

[bib0225] Laurenson Y.C.S.M., Kyriazakis I., Forbes A.B., Bishop S.C. (2012). Exploration of the epidemiological consequences of resistance to gastro-intestinal parasitism and grazing management of sheep through a mathematical model. Vet. Parasitol..

[bib0230] Laurenson Y.C.S.M., Kyriazakis I., Bishop S.C. (2012). In silico exploration of the impact of pasture larvae contamination and anthelmintic treatment on genetic parameter estimates for parasite resistance in grazing sheep. J. Anim. Sci..

[bib0235] Laurenson Y.C.S.M., Bishop S.C., Forbes A.B., Kyriazakis I. (2013). Modelling the short- and long-term impacts of drenching frequency and targeted selective treatment on the performance of grazing lambs and the emergence of anthelmintic resistance. Parasitology.

[bib0240] Laurenson Y.C.S.M., Kyriazakis I., Bishop S.C. (2013). Can we use genetic and genomic approaches to identify candidate animals for targeted selective treatment. Vet. Parasitol..

[bib0245] Li R.W., Hou Y., Li C., Gasbarre L.C. (2010). Localized complement activation in the development of protective immunity against *Ostertagia ostertagi* infections in cattle. Vet. Parasitol..

[bib0250] Louie K., Vlassoff A., Mackay A. (2005). Nematode parasites of sheep: extension of a simple model to include host variability. Parasitology.

[bib0255] MATLAB and Statistics Toolbox Release 2012, The MathWorks, Inc., Natick, Massachusetts, United States.

[bib0260] Mackinnon M., Meyer K., Hetzel D.J. (1991). Genetic variation and covariation for growth: parasite resistance and heat tolerance in tropical cattle. Livest. Prod. Sci..

[bib0265] Mansour M.M., Rowan T.G., Dixon J.B., Carter S.D. (1991). Immune modulation by *Ostertagia ostertagi* and the effects of diet. Vet. Parasitol..

[bib0270] Mansour M.M., Dixon J.B., Rowan T.G., Carter S.D. (1992). Modulation of calf immune responses by *Ostertagia ostertagi*: the effect of diet during trickle infection. Vet. Immunol. Immunopathol..

[bib0275] Michel J.F., Sinclair I.J. (1969). The effect of cortisone on the worm burdens of calves infected daily with *Ostertagia ostertagi*. Parasitology.

[bib0280] Michel J.F., Lancaster M.B., Hong C. (1978). The length of Ostertagia ostertagi in populations of uniform age. Int. J. Parasitol..

[bib0285] Michel J.F. (1969). Some observations on the worm burdens of calves infected daily with *Ostertagia ostertagi*. Parasitology.

[bib0290] Michel J.F. (1970). The regulation of populations of *Ostertagia ostertagi* in calves. Parasitology.

[bib0295] O’shaughnessy J., Earley B., Mee J.F., Doherty M.L., Crosson P., Barrett D., Prendiville R., Macrelli M., de Waal T. (2014). Detection of anthelmintic resistance on two Irish beef research farms. Vet. Rec..

[bib0300] Parkins J.J., Holmes P.H. (1989). Effects of gastrointestinal helminth parasites on ruminant nutrition. Nutr. Res. Rev..

[bib0305] Phillips C.J.C. (2010). Principles of Cattle Production.

[bib0310] Ploeger H.W., Kloosterman F.W., Rietveld A. (1995). Acquired immunity against *Cooperia* spp. and *Ostertagia* spp. in calves: effect of level of exposure and timing of the midsummer increase. Vet. Parasitol..

[bib0315] Prada Jiménez de Cisneros J., Stear M.J., Mair C., Singleton D., Stefan T., Stear A., Marion G., Matthews L. (2014). An explicit immunogenetic model of gastrointestinal nematode infection in sheep. J. R. Soc. Interface.

[bib0320] Prakash M. (2009). Introduction to Veterinary Genetics: Genetics Inbreeding and Crossbreeding.

[bib0325] Rose H., Rinaldi L., Bosco A., Mavrot F., de Waal T., Skuce P., Charlier J., Torgerson P.R., Hertzberg H., Hendrickx G., Vercruysse J., Morgan E.R. (2015). Widespread anthelmintic resistance in European farmed ruminants: a systematic review. Vet. Rec..

[bib0330] Saltelli A., Annoni P., Azzini I., Campolongo F., Ratto M., Tarantola S. (2010). Variance based sensitivity analysis of model output. Design and estimator for the total sensitivity index. Comput. Phys. Commun..

[bib0335] Sandberg F.B., Emmans G.C., Kyriazakis I. (2006). A model for predicting feed intake of growing animals during exposure to pathogens. J. Anim. Sci..

[bib0340] Satrija F., Nansen P. (1993). Experimental concurrent infections with *Ostertagia ostertagi* and *Cooperia oncophora* in the calf. Res. Vet. Sci..

[bib0345] Scott P.R., Penny C.D., Macrae A. (2011). Cattle Medicine.

[bib0350] Skuce P.J., Morgan E.R., van Dijk J., Mitchell M. (2013). Animal health aspects of adaptation to climate change: beating the heat and parasites in a warming Europe. Animal.

[bib0355] Smith G., Grenfell B.T. (1985). The population biology of *Ostertagia ostertagi*. Parasitol. Today.

[bib0360] Smith G., Grenfell B.T. (1994). Modelling of parasite populations: gastrointestinal nematode models. Vet. Parasitol..

[bib0365] Smith G., Grenfell B.T., Anderson R.M., Beddington J. (1987). Population biology of *Ostertagia ostertagi* and anthelmintic strategies against ostertagiasis in calves. Parasitology.

[bib0370] Smith G. (1997). The economics of parasite control: obstacles to creating reliable models. Vet. Parasitol..

[bib0375] Stear M.J., Bishop S.C. (1999). The curvilinear relationship between worm length and fecundity of *Teladorsagia circumcincta*. Int. J. Parasitol..

[bib0380] Stear M.J., Bishop S.C., Doligalska M., Duncan J.L., Holmes P.H., Irvine J., McCririe L., McKellar Q.A., Sinski E., Murray M. (1995). Regulation of egg production, worm burden, worm length and worm fecundity by host responses in sheep infected with *Ostertagia circumcincta*. Parasite Immunol..

[bib0385] Sutherland I.A., Leathwick D.M. (2011). Anthelmintic resistance in nematode parasites of cattle: a global issue?. Trends Parasitol..

[bib0390] Symeou V., Leinonen I., Kyriazakis I. (2014). Modelling phosphorus intake, digestion, retention and excretion in growing and finishing pig: model evaluation. Animal.

[bib0395] Szyszka O., Kyriazakis I. (2013). What is the relationship between level of infection and sickness behaviour in cattle?. Appl. Anim. Behav. Sci..

[bib0400] Taylor L.M., Parkins J.J., Armour J., Holmes K., Bairden K., Ibarra-silva A.M., Salman S.K., McWilliams P.N. (1989). Pathophysiological and Parasitological studies on *Ostertagi ostertagi* infections in calves. Res. Vet. Sci..

[bib0405] Tisdell C.A., Harrison S.R., Ramsay G.C. (1999). The economic impacts of endemic diseases and disease control programmes. Rev. Sci. Technol..

[bib0410] Todd D.L., Woolliams J.A., Roughsedge T. (2011). Gene flow in a national cross-breeding beef population. Animal.

[bib0415] Torgerson P.R., Paul M., Lewis F.I. (2012). The contribution of simple random sampling to observed variations in faecal egg counts. Vet. Parasitol..

[bib0420] Vagenas D., Bishop S.C., Kyriazakis I. (2007). A model to account for the consequences of host nutrition on the outcome of gastrointestinal parasitism in sheep: logic and concepts. Parasitology.

[bib0425] Vagenas D., Bishop S.C., Kyriazakis I. (2007). A model to account for the consequences of host nutrition on the outcome of gastrointestinal parasitism in sheep: model evaluation. Parasitology.

[bib0430] Vagenas D., Doeschl-Wilson A., Bishop S.C., Kyriazakis I. (2007). In silico exploration of the effects of host genotype and nutrition on the genetic parameters of lambs challenged with gastrointestinal parasites. Int. J. Parasitol..

[bib0435] Van Bruchem J.M.W.B., Lammers-Weinhoven S.C., Bangma G.A. (1991). Intake, rumination, reticulo-rumen fluid and particle kinetics, and faecal particle size in heifers and cattle fed on grass hay and wilted grass silage. Livest. Prod. Sci..

[bib0440] Vercruysse J., Claerebout E. (1997). Immunity development against *Ostertagia ostertagi* and other gastrointestinal nematodes in cattle. Vet. Parasitol..

[bib0445] Verschave S.H., Vercruysse J., Claerebout E., Rose H., Morgan E.R., Charlier J. (2014). The parasitic phase of *Ostertagia ostertagi*: quantification of the main life history traits through systematic review and meta-analysis. Int. J. Parasitol..

[bib0450] Viney M.E., Riley E.M., Buchanan K.L. (2005). Optimal immune responses: immunocompetence revisited. Trends Ecol. Evol..

[bib0455] Ward C.J. (2006). Mathematical models to assess strategies for the control of gastrointestinal roundworms in cattle 1. Construction. Vet. Parasitol..

[bib0460] Ward C.J. (2006). Mathematical models to assess strategies for the control of gastrointestinal roundworms in cattle 2. Validation. Vet. Parasitol..

[bib0465] Wellock I.J., Emmans G.C., Kyriazakis I. (2004). Describing and predicting potential growth in the pig. Anim. Sci..

[bib0470] Wiggin C.J., Gibbs H.C. (1989). Studies of the immunomodulatory effects of low-level infection with *Ostertagia ostertagi* in calves. Am. J. Vet. Res..

[bib0475] Xiao L., Gibbs H.C. (1992). Nutritional and pathophysiologic effects of clinically apparent and subclinical infections of *Ostertagia ostertagi* in calves. Am. J. Vet. Res..

[bib0480] Young R., Anderson N. (1981). The ecology of the free-living stages of *Ostertagia ostertagi* in a winter rainfall region. Aust. J. Agric. Res..

