# Supplementary material for: A simulation model to investigate interactions between first season grazing calves and Ostertagia ostertagi
Source: Vet Parasitol. 2016 Aug 15;226:198–209. doi: 10.1016/j.vetpar.2016.05.001 (PMC4990062; doi:10.1016/j.vetpar.2016.05.001)
Supplement: Supplementary file 1 [file mmc1.docx]

## Supplementary Data S1

Previously published generic equations and relationships used as the basis for the model are presented below, along with more detailed justifications for determining intrinsic calf growth rate and body composition.

### Calf body composition

The live body weight of a calf consists of gut fill and empty body weight (EBW). EBW composition comprises of four main components; protein, lipid, ash and water. The methodology for the calculation of the mature body composition is presented below.

The calf intrinsic growth rate parameter (B) can be calculated by the method of [Kyriazakis and Emmans (1990](#_ENREF_23)) as:

|  | $B\left( t_{1}-t_{2} \right)=-ln (-\ln\frac{w_{2}}{w_{m}})+\ln(-\ln\frac{w_{1}}{w_{m}})$  (day^-1^) | (A.1) |
| --- | --- | --- |

Where $B$ is the intrinsic growth rate, $w_{1}$is the calf body weight recorded at time $t_{1}$ and $w_{2}$is the weight recorded at time $t_{2}$; $w_{m}$is the calf body weight at maturity.

Calf ’target’ live body weights ($w$) of the genotype used were taken from EBLEX (2005). An average Limousin x Holstein-Friesian bull is expected to reach a mature weight ($w_{m}$) of 1000kg ([The British Limousin Cattle Society,](#_ENREF_12) 2010). Steers are expected to achieve a $w_{m}$ of 800kg, consistent with [AFRC ( 1993](#_ENREF_1))

Using the above values an intrinsic growth rate (B) of 0.00711 day^-1^ was calculated. From this a mature protein content ($P_{M}$) of 106kg was predicted, assuming a constant B* (B x Pm) value of 0.025 (Emmans, 1997) :

| $P_{M}= \sqrt[0.27]{\left( \frac{B^{*}}{B} \right)}$  (kg) | (A.2) |
| --- | --- |

where $P_{M}$ is the mature protein content of the calf (kg) and B* is a constant relationship between mature protein content and growth across mammals (Emmans, 1997).

Mature water and ash content were both assumed to be functions of the body protein content (Emmans and Kyriazakis, 1997), hence the remaining components of the mature empty body weight (${EBW_{M}}$) can be assumed to be lipid. The gutfill of a steer can range from 5-25% depending on the quality of the feed available ([Louw, 1988](#_ENREF_27); NCR, 2001). For an average quality feed the EBW was assumed to be 85% of body weight (Van Souest, 1994; Williams and Jenkins, 1997); hence a Limousin X Holstein-Friesian steer was assumed to have $EBW_{M}$ of 680kg. The mature lipid content was calculated at 207kg; this is consistent with literature reports of EBW fat percentages at slaughter of 25 and 30% $w_{m}$ (Williams and Jenkins, 1997).

### Basic Intrinsic Growth Model

The intrinsic (maximum) body protein growth ($\Delta PGrow{th}_{max}$ ) was estimated by Emmans (1997) as:

| $\Delta PGrow{th}_{max}=P.B.\ln\left( \frac{P_{M}}{P} \right)$  (kg/day) | (A.3) |
| --- | --- |

where $P$ is the current protein mass.

The daily ash accretion ($\Delta Ash$ ) was estimated by Emmans and Kyriazakis (1997) as:

| $\Delta Ash=0.211.\Delta PGrowth$  (kg/day) | (A.4) |
| --- | --- |

and the daily water accretion ($\Delta Water$ ) as:

| $\Delta Water=2.65.\Delta PGrowth\left( \frac{P}{P_{M}} \right)^{-0.185}$  (kg/day) | (A.5) |
| --- | --- |

The desired daily lipid deposition ($\Delta PLipid_{des}$) was estimated by Emmans and Kyriazakis (1999) as:

| $\Delta PLipid_{des}=\Delta PGrow{th}_{max}.\left( \frac{L_{M}}{P_{M}} \right).{d .\left( \frac{P}{P_{M}} \right)}^{(d-1)}$  (kg/day) | (A.6) |
| --- | --- |

where $L_{M}$ is the lipid at maturity (kg) and $d$ is given as (Emmans, 1997):

$$d=1.46.\left( \frac{L_{M}}{P_{M}} \right)^{0.23}$$

(A.7)

The gutfill (GF) of the calf depends largely on the food intake and the Metabolisable Energy (ME) content of the feed (MJ/kg DM) (Coffey et al., 2001):

| $GF=FI\left( 11-\frac{7.ME}{15} \right)$  (kg/day) | (A.8) |
| --- | --- |

where $FI$ is the food intake (kg DM/day.)

### Resource requirement and Food Intake

The maintenance requirements for protein and energy, ${PR}_{maint}{and ER}_{maint}$ respectively, were both estimated as functions of P and Pm (Emmans & Fisher, 1986; Wellock et al., 2003):

| ${PR}_{maint}=0.004 \frac{P}{{P_{M}}^{0.27}}$  (kg/day)  ${ER}_{maint}=1.63 \frac{P}{{P_{M}}^{0.27}}$  (MJ/day) | (A.9)  (A.10) |
| --- | --- |

The growth requirement for protein (${PR}_{growth}$) was estimated by Wellock et al. (2003):

| ${PR}_{growth}= \frac{\Delta PGrow{th}_{max}}{ep}$  (kg/day) | (A.11) |
| --- | --- |

where *ep* is the efficiency of protein deposition, assumed to be 0.26 (AFRC, 1993).

The growth requirement for energy (${ER}_{growth}$) was estimated by Wellock et al (2003):

| ${ER}_{growth}=\left( bl.\Delta PLipid_{des} \right)+(bp.\Delta PGrow{th}_{max})$  (MJ/day) | (A.12) |
| --- | --- |

Where *bl* is the energetic cost per kg of lipid deposition of 56 MJ/kg (Emmans, 1994) and *bp* is the energetic cost of per kg protein deposition of 50 MJ/kg (Emmans, 1994).

The desired food intake to meet the total energy requirements of the calf (${FI}_{E}$) was estimated as (Vagenas et al., 2007a):

| ${FI}_{E}=\frac{ER}{EEC}$  (kg DM/day) | (A.13) |
| --- | --- |

where$ER$ is the total daily energy requirement and $EEC$ is the effective energy content of the feed given as (Emmans, 1994):

| $EEC=1.15ME-3.84-4.67(0.9CP-0.032)$  (MJ/kg DM) | (A.14) |
| --- | --- |

where *ME* is the metabolisable energy content of the feed (MJ/kg DM), and *CP* is the crude protein content of the feed (g/kg DM).The desired food intake to meet the total protein requirements of the calf (${FI}_{P}$) was estimated by Laurenson et al (2011) as:

| ${FI}_{P}=\frac{PR}{0.9CP-0.032}$  (kg DM/day) | (A.15) |
| --- | --- |

where$PR$ is the total daily protein requirement and $CP$ is the crude protein content of the feed (g/kg DM).

### Constrained Resources

Constrained food intake (CFI) was defined as follows (Lewis et al., 2004):

$CFI=\frac{CAP}{0.93-\left( \frac{ME}{15.58} \right)}$

(kg/day) (A.16)

where CAP is the capacity of the animal for daily indigestible organic matter (kg) and ME is the metabolisable energy content of the feed (MJ/kg DM).

The capacity of the animal for daily indigestible organic matter (*CAP*) was estimated as the smaller of: *CAP*= 0·0223·*BW* or *CAP*= 0·0223·0·51·*BW_M_* (kg/day) where BW is the current body weight of the calf (kg) and *BW_M_* is the body weight of the calf at maturity (kg).

### Allocation of Nutrient resources

The daily lipid deposited was described by the following equation (Vagenas et al., 2007a).

| $\Delta Lipid=\frac{(\left( FI.EEC \right)-E_{maint}-E_{protein})}{bl}$  (kg/day) | (A.17) |
| --- | --- |

where$E_{maint}$ is the energy given to maintenance, $E_{protein}$ is the energy given to protein growth ($bp.\Delta PGrow{th}_{max}$).

When there are insufficient resources, and in the case of lipid catabolism, the *bl*  parameter was replaced by the heat combustion of lipid (${bp}_{c})$ assumed to be 39 MJ/kg (AFRC, 1993).

Labile protein (maximum amount of protein the animal can mobilize from its body) was defined by (Houdijk et al., 2001; Sykes, 2000):

| $P_{Labile}=0.2.P_{max}$  (kg) | (A.18) |
| --- | --- |

where $P_{max}$ is the maximum achieved body protein content (kg).

The baseline lipid level (the minimum body lipid level required for survival) is defined as (Vagenas et al., 2007a):

| $L_{Base}=0.2. P$  (kg) | (A.19) |
| --- | --- |

### Protein Loss

The protein loss associated with both larval burden and worm mass was described in the paper. This loss is prior to any immune response and hence the protein loss was re-calculated following this consideration. The actual protein loss caused by larval burden after considering this effect has been accounted for ($PLB$) (Vagenas et al., 2007a):

| $PLB={PLB}_{Pot}\left( \frac{{PLB}_{Pot}. e^{-K_{Imm}.{PRQ}_{Imm}}}{{Ploss}_{max}} \right)^{\left( \frac{{PAC}_{Imm}}{\left( {PAC}_{Imm} \right)_{max}} \right)}$  (kg/day) | (A.20) |
| --- | --- |

where ${PRQ}_{Imm}$is the protein required by the immune response, ${PAC}_{Imm}$ is the protein allocated to the immune response, (*PAC_Imm_*)*_max_* is the maximum protein allocated to immunity (0.2*P_maint_*), *K_Imm_* is an the immune exponent detailed in equation (A.21), *PLB_Pot_* is the potential protein associated with larval burden as described in the paper (equation 12), and *Ploss_max_* is the maximum protein loss (0.5kg/d) as described in the paper (equation 14).

Protein loss has been calculated prior to consideration of the immune response. The actual protein loss caused by worm mass after considering this effect has been accounted for ($PWM$) (Vagenas et al., 2007a):

| $PWM={PWM}_{Pot}\left( \frac{{PWM}_{Pot}. e^{-K_{Imm}.{PRQ}_{Imm}}}{{Ploss}_{max}} \right)^{\left( \frac{{PAC}_{Imm}}{\left( {PAC}_{Imm} \right)_{max}} \right)}$  (kg/day) | (A.21) |
| --- | --- |

where *PWM_Pot_* is the potential protein associated with worm mass as described in the paper (equation 13).

The potential protein loss was affected by the immune exponent ($K_{Imm}$) (Vagenas et al., 2007a):

| $K_{Imm}=\frac{\ln\left( \frac{{Ploss}_{target}}{{Ploss}_{max}} \right)}{\left( {PAC}_{Imm} \right)_{max}}$ | (A.22) |
| --- | --- |

where ${Ploss}_{target}$ is the value at which the animal stops allocating protein to immunity (0.001kg/d).

### Immune Requirements

The protein required for immunity for larval burden ($PRQLB_{Imm}$) is estimated by Vagenas (2007a) as:

$$PRQLB_{Imm}={{(PAC}_{Imm})}_{max}\cdot\frac{\ln\left( \frac{{Ploss}_{target}}{{PLB}_{pot}} \right)}{\ln\left( \frac{{Ploss}_{target}}{{PLoss}_{max}} \right)}$$

(kg/day) (A.23)

where ${Ploss}_{target}$ is the minimum damage for which there is no immune response (0·0001(Vagenas et al., 2007a;2007b))

The protein required for immunity for worm mass ($PRQWM_{Imm}$) was also estimated by Vagenas et al. (2007a) as:

$$PRQWM_{Imm}=- \frac{\ln\left( \frac{{Ploss}_{target}}{PWM} \right)}{-K_{Imm}}$$

(kg/day) (A.24)

### Protein Partitioning

Protein allocated to growth depends on the requirements for both immunity (*PR_Imm_*) and growth (*PR_Growth_*), the proportion of protein allocated to growth (${PAC}_{Growth}$) is given as (Vagenas et al., 2007a):

| ${PAC}_{Growth}=\frac{{PR}_{Growth}}{{{PR}_{Growth}+PR}_{Imm}}$  (kg/day) | (A.25) |
| --- | --- |

The proportion of protein allocated to immunity (${PAC}_{Imm}$) (Vagenas et al., 2007a):

| ${PAC}_{Imm}=\frac{{PR}_{Imm}}{{{PR}_{Growth}+PR}_{Imm}}$  (kg/day) | (A.26) |
| --- | --- |

The efficiency of metabolisable protein use in immunity is considered to be 0.59 (Laurenson et al., 2011)

References

Coffey M.P., Emmans G.C. and Brotherstone S., Genetic evaluation of dairy bulls for energy balance traits using random regression, *Anim. Sci.* **73**, 2001, 29–40

Emmans G.C., Effective energy– a concept of energy-utilization applied across species, *Brit. J. Nutr.* **71**, 1994, 801–821

Emmans G.C. and Fisher C., Problems of nutritional theory, Nutritional Requirements and Nutritional Theory, 1986, Butterworths; London, 9–57

Emmans G.C. and Kyriazakis I., Growth and body composition, A Quantitative Biology of the Pig, 1999,CAB International; Wallingford, UK, 181–197

Houdijk J.M., Jessop N.S. and Kyriazakis I., Nutrient partitioning between reproductive and immune functions in animals, *Proc. Nutr. Soc.* **60**, 2001, 515–525

Lewis R.M., Macfarlane J.M., Simm G., et al., Effects of food quality on growth and carcass composition in lambs of two breeds and their cross, *Anim Sci.* **78**, 2004, 355–367

Louw B.P., The influence of loss and gain of body mass on ovarian activity in beef cows, *S. Afr. J.* *Anim. Sci.* **18**, 1988, 1–7

NRC, Energy, Nutrient Requirements of Dairy Cattle, 2001, National Academies Press, 13–28

The British Limousin Cattle Society, 2010 <http://limousin.co.uk/the-breed/breed-standard/> (accessed: April 2016).

Sykes A.R., Environmental effects on animal production: the nutritional demands of nematode parasite exposure in sheep, *Asian Australas. J. Anim.* **13**, 2000, 343–350

Van Souest P.J., Body size and limitations of ruminants, Nutritional Ecology of the Ruminant, 1994, Conrell university Press; Ithaca and London, 40–56

Wellock I.J., Emmans G.C. and Kyriazakis I., Modelling the effects of thermal environment and dietary composition on pig performance: model logic and concepts, *Anim. Sci.* **77**, 2003, 255–266

Williams C.B. and Jenkins T.G., Predicting empty body composition and composition of empty body weight changes in mature cattle, *Agric. Syst.* **53**, 1997, 1–25.
