# Supplementary material for: A simulation model to investigate interactions between first season grazing calves and Ostertagia ostertagi
Source: Vet Parasitol. 2016 Aug 15;226:198–209. doi: 10.1016/j.vetpar.2016.05.001 (PMC4990062; doi:10.1016/j.vetpar.2016.05.001)
Supplement: Supplementary file 2 [file mmc2.docx]

## Supplementary Data S2

### The consequences of different modes of infection

As many experiments, for practical reasons, challenge animals with single or weekly doses of larvae, the model was investigated for its behaviour under such scenarios. The worm burdens of a single calf infected over the course a three week period with a total of 210,000 *O. ostertagi* larvae administered either daily (10,000 L_3_ per day trickle challenge), or in three weekly doses of 70,000 L_3_, or as a single dose at the start of the period are shown in figure S1.

The single infection resulted in a higher and an earlier peak worm burdens, which was observed on d24 post infection (pi) (figure S1.a); the peak worm burdens for the weekly and trickle infections were observed on d37 and 40 pi respectively. This was a reflection of both the mode of infection and the associated development of the immune response. Worm burdens started to decline at a faster rate for single infections. Worm burdens declined to negligible levels for all modes of administration, as no new larvae were administered after week 3 pi.

The worm burden patterns of the different modes of administration were reflected in the numbers of total egg outputs produced (figure S1.b), with single infections resulting in a higher and earlier maximum total egg output. Differences in the patterns of worm burden and total egg outputs reflect the impact of immunity of worm fecundity and the density-dependent effects on fecundity.

A reduction in food intake was observed for all modes of larval administration; the point of maximum intake reduction was observed to be earlier and recovery to be slightly faster for infections administered through fewer doses (figure S1.c). The recovery began at d29, 36 and 39 pi for single, weekly and daily modes of infection respectively; this corresponded closely to the peak timing of the worm burdens, as the model assumes for both to be dependent on the development of the immune response. Food intake remains slightly below that of a healthy host for all methods of administration, due to the slow continual development of immunity.

The reductions in calf bodyweight, comparative to the uninfected control calf, for the different modes of larval administration are shown in figure S1.d. Although the reduction in bodyweight reached its maximum value earlier in the single infection, the final bodyweight loss was more pronounced in trickle infections.
