# Supplementary material for: A simulation model to investigate interactions between first season grazing calves and Ostertagia ostertagi
Source: Vet Parasitol. 2016 Aug 15;226:198–209. doi: 10.1016/j.vetpar.2016.05.001 (PMC4990062; doi:10.1016/j.vetpar.2016.05.001)
Supplement: Supplementary file 3 [file mmc3.docx]

## Supplementary Data S3

### Additional model validation

Additional validations of the model against the published experiments of Michel (1969; Experiment B) (Fig S2), Claerebout et al. (1996) (Fig S3), Forbes et al. (2009) (Fig S4), Hilderson et al. (1993) (Fig S5), Hilderson et al. (1995) (Fig S6), Mansour et al. (1992) (Fig S7) and Xiao and Gibb (1992) (Fig S8) are presented below. The outcomes of statistical analyses used to assess goodness-of-fit between predictions and observed and experimental results are presented on Table 2 of the main paper. In most cases the comparsion between experimental and model observations for worm burdens and FEC showed a similar patterns, as reflected by the high positive correlation coefficients between the two.
