# Supplementary material for: A simulation model to investigate interactions between first season grazing calves and Ostertagia ostertagi
Source: Vet Parasitol. 2016 Aug 15;226:198–209. doi: 10.1016/j.vetpar.2016.05.001 (PMC4990062; doi:10.1016/j.vetpar.2016.05.001)
Supplement: Supplementary file 4 [file mmc4.docx]

## Supplementary Table S1

### Sensitivity analysis: P values

**Table S1**: Tables of P values for ANOVA tests conducted for the 5 key outputs (peak worm burden, time of the peak worm burden (days), the peak total egg count (eggs/d), the maximum reduction in food intake (%) and the peak body weight loss) and the significance of 12 key model parameters as described in the text. The values are given for the 3 infection levels explored of A)3500 L_3_ larvae/day, B)7000 L_3_ larvae/day and C)14,000 L_3_ larvae/day. The significant parameters are reported in the main text.

| **Challenge Dose (L_3_/day)** | **Parameter** | **Peak worm burden (worms)** | **Time of peak worm burden (days)** | **Peak total egg count (eggs/d)** | **Maximum reduction in food intake (%)** | **Peak BW loss (kg)** |
| --- | --- | --- | --- | --- | --- | --- |
| **3500** | **Max.estabmort** | 0.000 | 0.116 | 0.000 | 0.867 | 0.037 |
|  | **Minestabmort** | 0.885 | 0.831 | 0.671 | 0.353 | 0.573 |
|  | **Rate.estabmort** | 0.000 | 0.000 | 0.000 | 0.000 | 0.000 |
|  | **Max.mortality** | 0.240 | 0.003 | 0.022 | 0.360 | 0.465 |
|  | **Min.mortality** | 0.085 | 0.500 | 0.023 | 0.245 | 0.443 |
|  | **Rate.mortality** | 0.000 | 0.000 | 0.000 | 0.745 | 0.080 |
|  | **Max.fecundity** | 0.029 | 0.200 | 0.000 | 0.580 | 0.648 |
|  | **Min.fecundity** | 0.777 | 0.867 | 0.869 | 0.865 | 0.947 |
|  | **Rate.fecundity** | 0.296 | 0.653 | 0.263 | 0.237 | 0.762 |
|  | **Rate.anorexia** | 0.489 | 0.502 | 0.639 | 0.000 | 0.921 |
|  | **PlossWM** | 0.952 | 0.996 | 0.937 | 0.985 | 0.000 |
|  | **PlossLM** | 0.070 | 0.338 | 0.434 | 0.823 | 0.000 |
| **7000** | **Max.estabmort** | 0.000 | 0.210 | 0.000 | 0.865 | 0.146 |
|  | **Minestabmort** | 0.879 | 0.811 | 0.650 | 0.347 | 0.421 |
|  | **Rate.estabmort** | 0.000 | 0.000 | 0.000 | 0.000 | 0.000 |
|  | **Max.mortality** | 0.224 | 0.000 | 0.015 | 0.356 | 0.971 |
|  | **Min.mortality** | 0.170 | 0.409 | 0.054 | 0.250 | 0.117 |
|  | **Rate.mortality** | 0.000 | 0.000 | 0.000 | 0.746 | 0.039 |
|  | **Max.fecundity** | 0.032 | 0.424 | 0.000 | 0.574 | 0.118 |
|  | **Min.fecundity** | 0.798 | 0.973 | 0.899 | 0.869 | 0.895 |
|  | **Rate.fecundity** | 0.293 | 0.645 | 0.229 | 0.236 | 0.573 |
|  | **Rate.anorexia** | 0.489 | 0.475 | 0.632 | 0.000 | 0.994 |
|  | **PlossWM** | 0.955 | 0.949 | 0.955 | 0.984 | 0.000 |
|  | **PlossLM** | 0.062 | 0.454 | 0.348 | 0.824 | 0.000 |
| **14,000** | **Max.estabmort** | 0.000 | 0.448 | 0.000 | 0.863 | 0.147 |
|  | **Minestabmort** | 0.872 | 0.517 | 0.609 | 0.347 | 0.774 |
|  | **Rate.estabmort** | 0.000 | 0.000 | 0.000 | 0.000 | 0.000 |
|  | **Max.mortality** | 0.184 | 0.000 | 0.007 | 0.352 | 0.457 |
|  | **Min.mortality** | 0.256 | 0.348 | 0.093 | 0.252 | 0.125 |
|  | **Rate.mortality** | 0.000 | 0.000 | 0.000 | 0.748 | 0.020 |
|  | **Max.fecundity** | 0.038 | 0.751 | 0.000 | 0.572 | 0.142 |
|  | **Min.fecundity** | 0.815 | 1.000 | 0.924 | 0.868 | 0.886 |
|  | **Rate.fecundity** | 0.279 | 0.786 | 0.187 | 0.239 | 0.700 |
|  | **Rate.anorexia** | 0.481 | 0.407 | 0.625 | 0.000 | 0.658 |
|  | **PlossWM** | 0.957 | 0.584 | 0.961 | 0.984 | 0.000 |
|  | **PlossLM** | 0.055 | 0.770 | 0.282 | 0.825 | 0.000 |
