# Supplementary figures and images for: A simulation model to investigate interactions between first season grazing calves and Ostertagia ostertagi
Source: Vet Parasitol. 2016 Aug 15;226:198–209. doi: 10.1016/j.vetpar.2016.05.001 (PMC4990062; doi:10.1016/j.vetpar.2016.05.001)

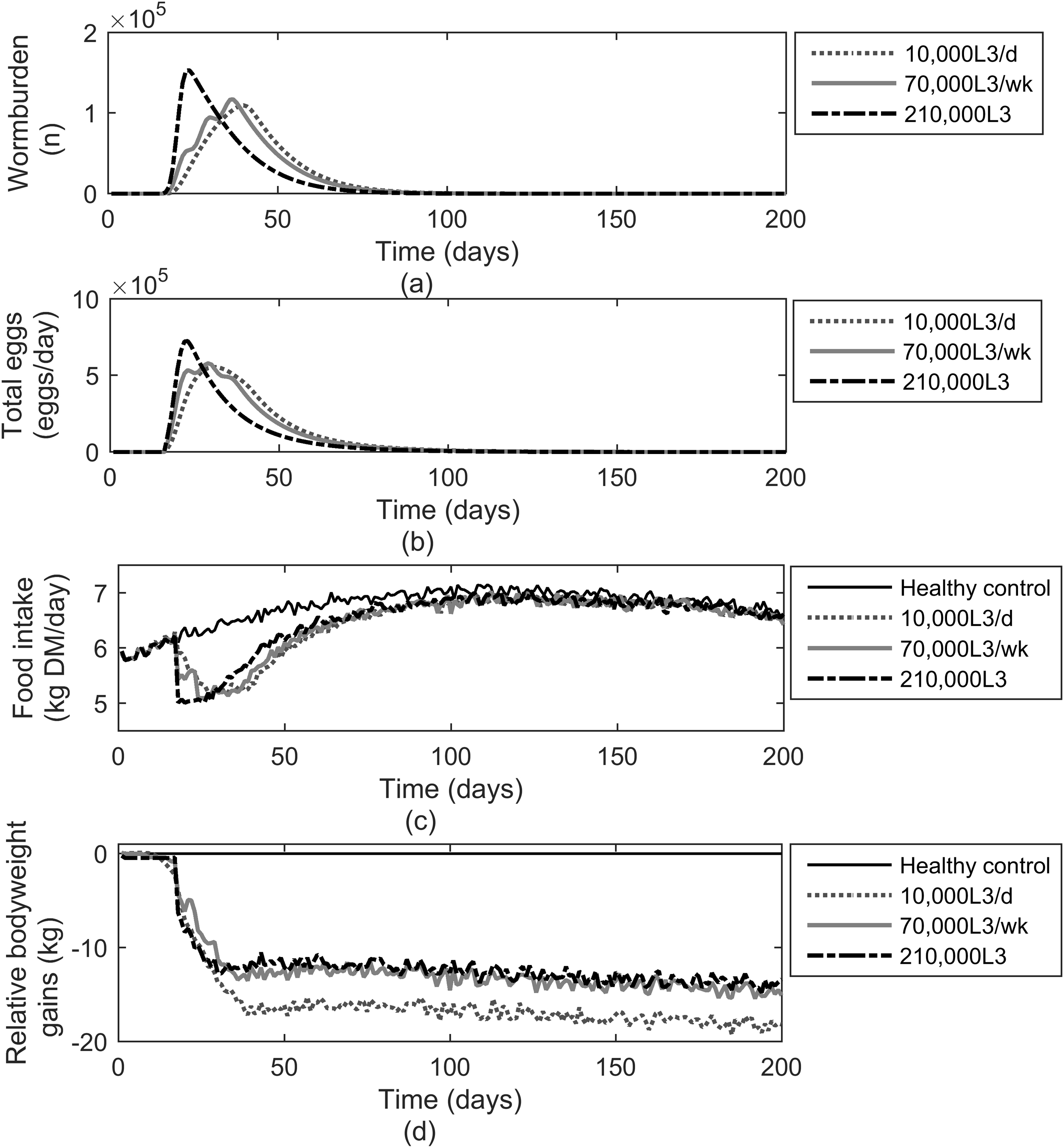

Supplement: Fig. S1 — Worm burden (a), daily faecal egg output (b), daily food intake (c) and total relative bodyweight loss (in comparison to uninfected controls, losses are cumulative over time) (d) incurred over time in calves given a total of 210,000 Ostertagia ostertagi larvae over three weeks administered either daily (10,000 per day trickle challenge), as three weekly doses of 70,000, or as a single dose at the start of the period. [file mmc5.jpg]

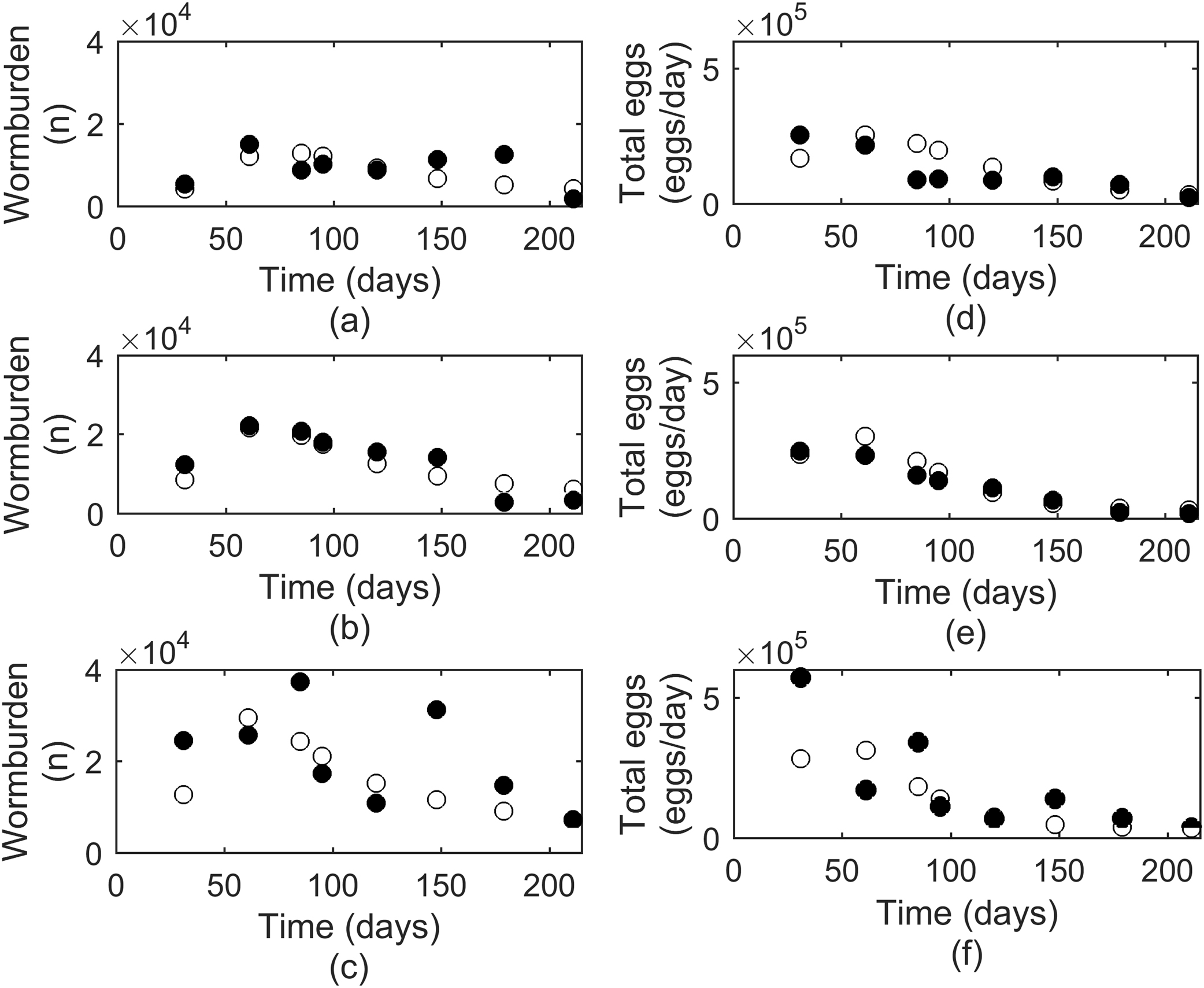

Supplement: Fig. S2 — A comparison of experimental observations (●) by Michel (1969) experiment B to simulated predictions (○) for worm burdens resulting from infection doses of (a) 500 larvae per day; (b) 1000 larvae per day; (c) 1500 larvae per day and total eggs per day resulting from infection levels of (d) 500 larvae per day; (e) 1000 larvae per day; (f) 1500 larvae per day. Each experimental data point is based on measurements from a single calf. [file mmc6.jpg]

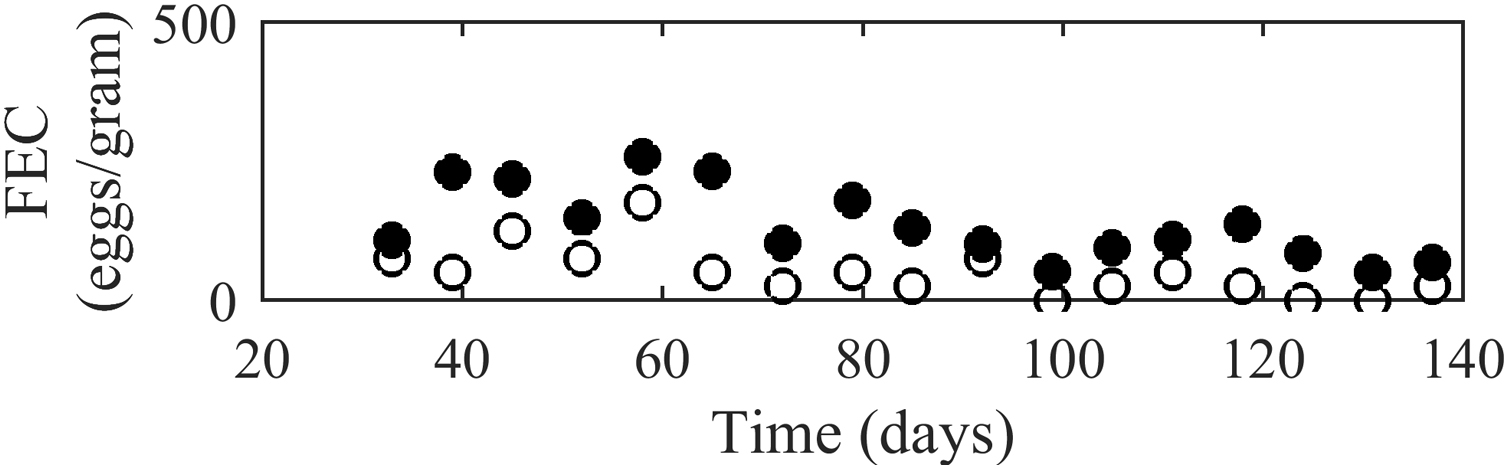

Supplement: Fig. S3 — A comparison of experimental observations (●) by Claerebout et al. (1996) to simulated predictions (○) for faecal egg outputs per gram of fresh faeces produced by an infection level of 20,000 larvae per week, administered in 3 doses, for 21 weeks. Each measurement was taken for 6 calves. [file mmc7.jpg]

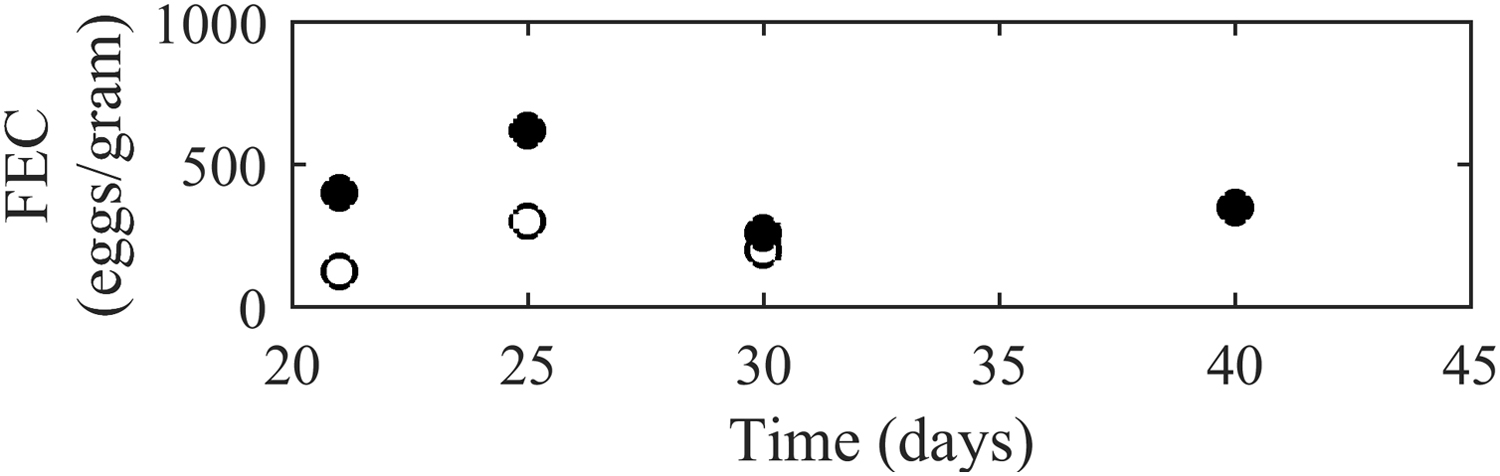

Supplement: Fig. S4 — A comparison of experimental observations (●) by Forbes et al. (2009) to simulated predictions (○) for faecal egg outputs per gram of fresh faeces produced by an infection level of 70,000 larvae per week, administered in 3 doses, for 8 weeks. Each measurement was taken for 5 calves. [file mmc8.jpg]

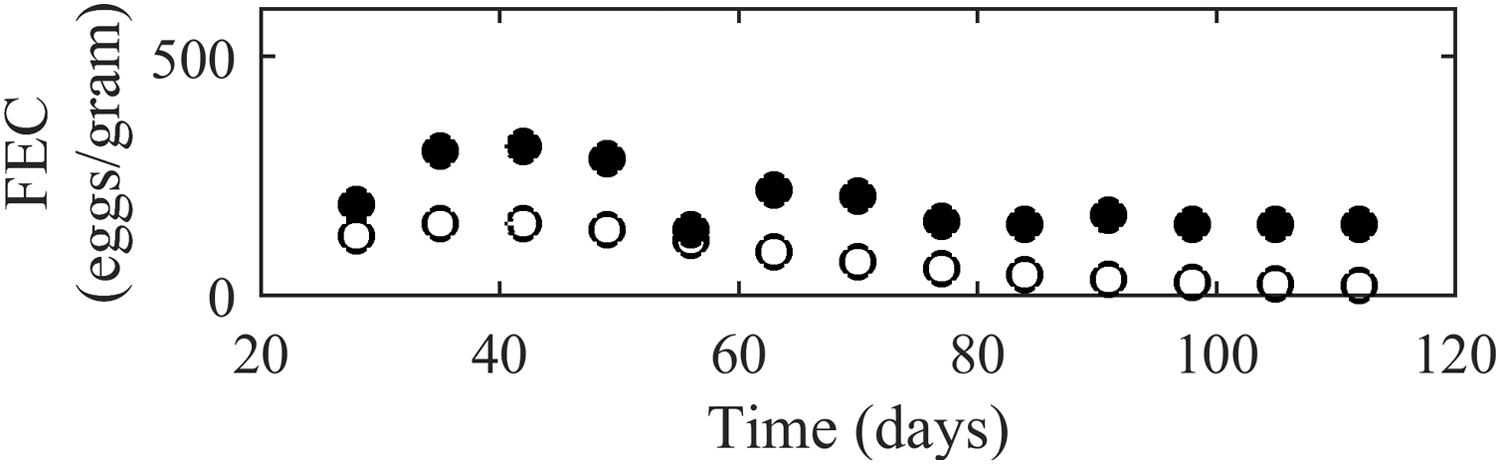

Supplement: Fig. S5 — A comparison of experimental observations (●) by Hilderson et al. (1993) to simulated predictions (○) for faecal egg outputs per gram of fresh faeces produced by infection levels of (a) 5000 larvae per week; (b) 10,000 larvae per week; (c) 20,000 larvae per week; (d) 40,000 larvae per week, all administered in 3 doses a week for 17 weeks. Each measurement was taken for 4 calves. [file mmc9.jpg]

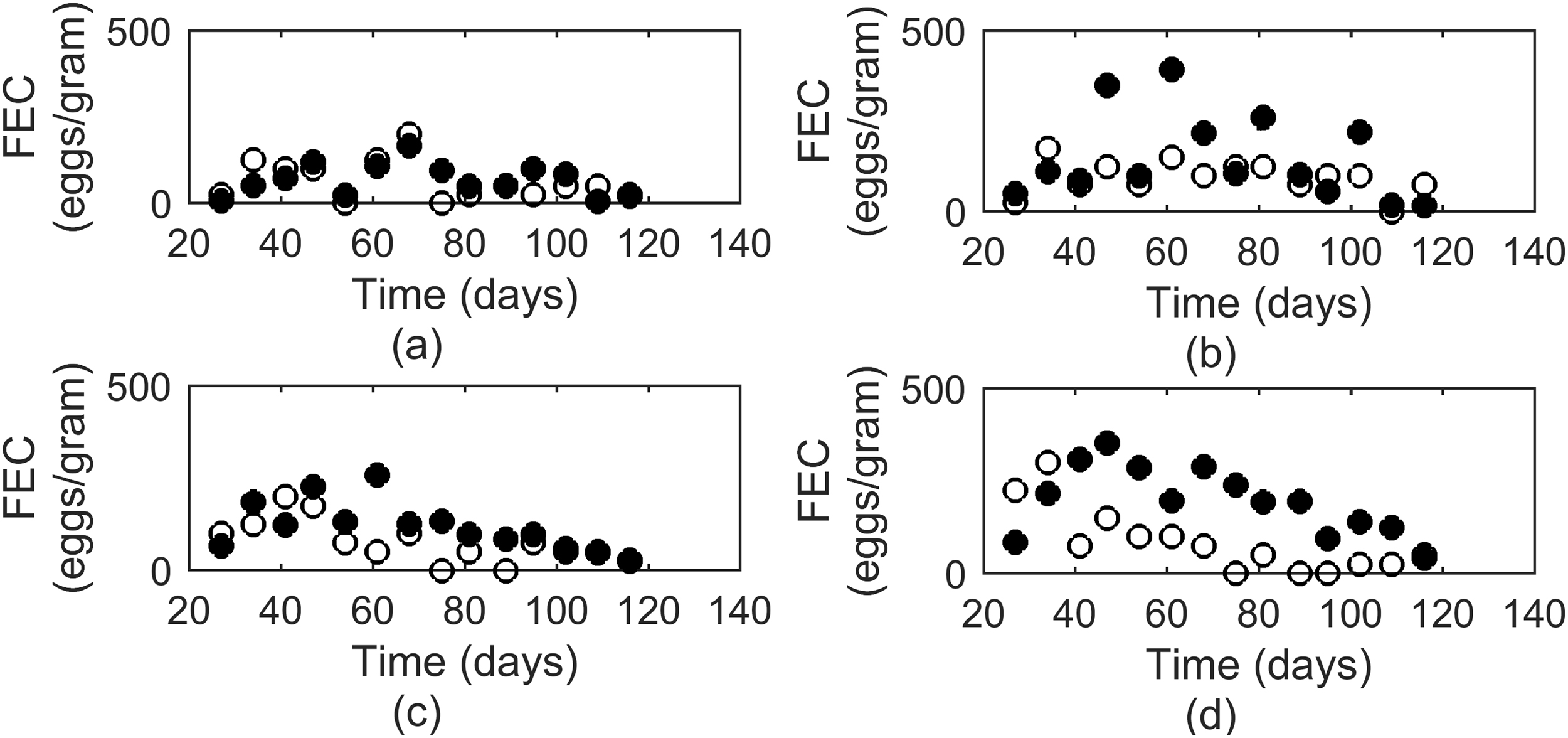

Supplement: Fig. S6 — A comparison of experimental observations (●) by Hilderson et al. (1995) to simulated predictions (○) for faecal egg outputs per gram of fresh faeces produced an infection level of 20,000 larvae per week, administered in 3 doses, for 17 weeks. Each measurement was taken for 5 calves. [file mmc10.jpg]

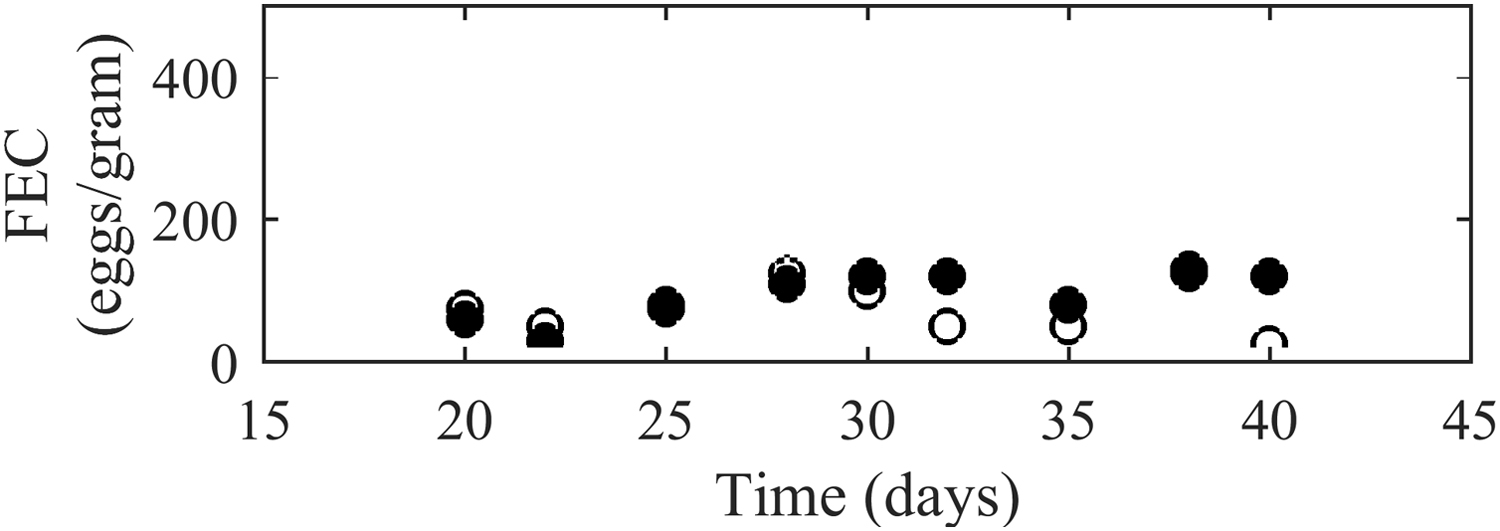

Supplement: Fig. S7 — A comparison of experimental observations (●) by Mansour et al. (1992) to simulated predictions (○) for faecal egg outputs per gram of fresh faeces produced by an infection level of 3000 larvae administered every other day for 6 weeks. Each measurement was taken for 6 calves. [file mmc11.jpg]

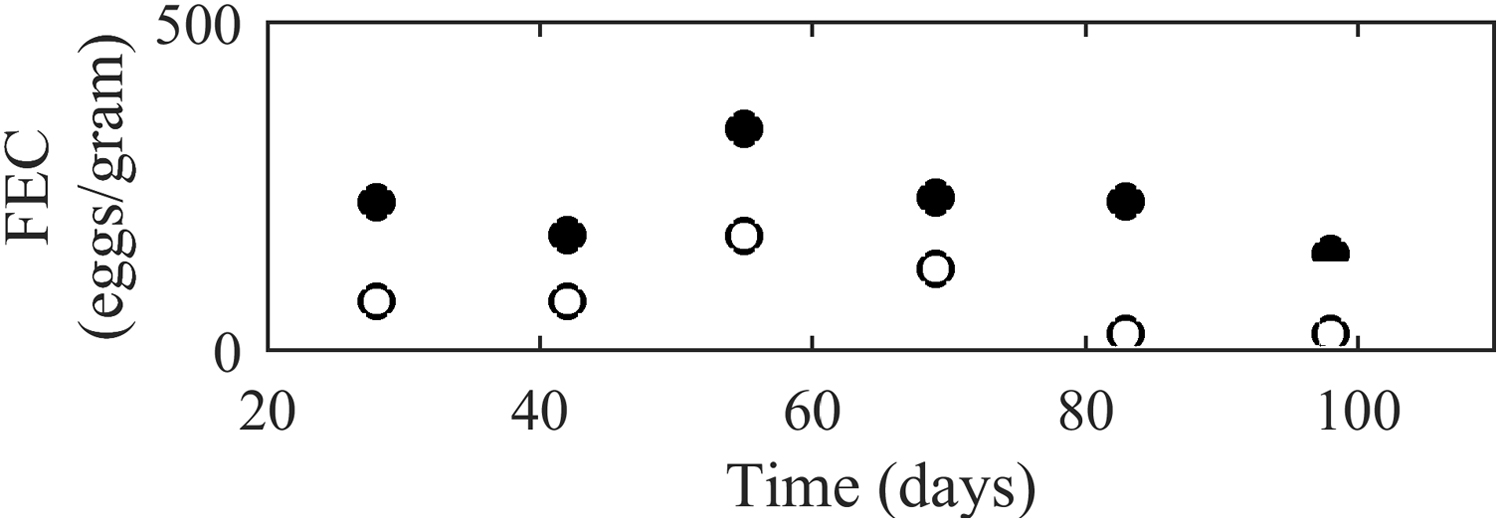

Supplement: Fig. S8 — A comparison of experimental observations (●) by Xiao and Gibbs (1992) to simulated predictions (○) for faecal egg outputs per gram of fresh faeces produced by a weekly infection of 10,000 larvae for 14 weeks. Each measurement was taken for 5 calves. [file mmc12.jpg]
